# Supplementary material for: Human cytomegalovirus forms phase-separated compartments at viral genomes to facilitate viral replication
Source: Cell Rep. 2022 Mar 8;38(10):110469. doi: 10.1016/j.celrep.2022.110469 (PMC8924372; doi:10.1016/j.celrep.2022.110469)
Supplement: Document S1. Figures S1–S5 [file mmc1.pdf]

Cell Reports, Volume *38*

Supplemental information

Human cytomegalovirus forms phase-separated compartments at viral genomes to facilitate viral replication

Enrico Caragliano, Stefano Bonazza, Giada Frascaroli, Jiajia Tang, Timothy K. Soh, Kay Grünewald, Jens B. Bosse, and Wolfram Brune

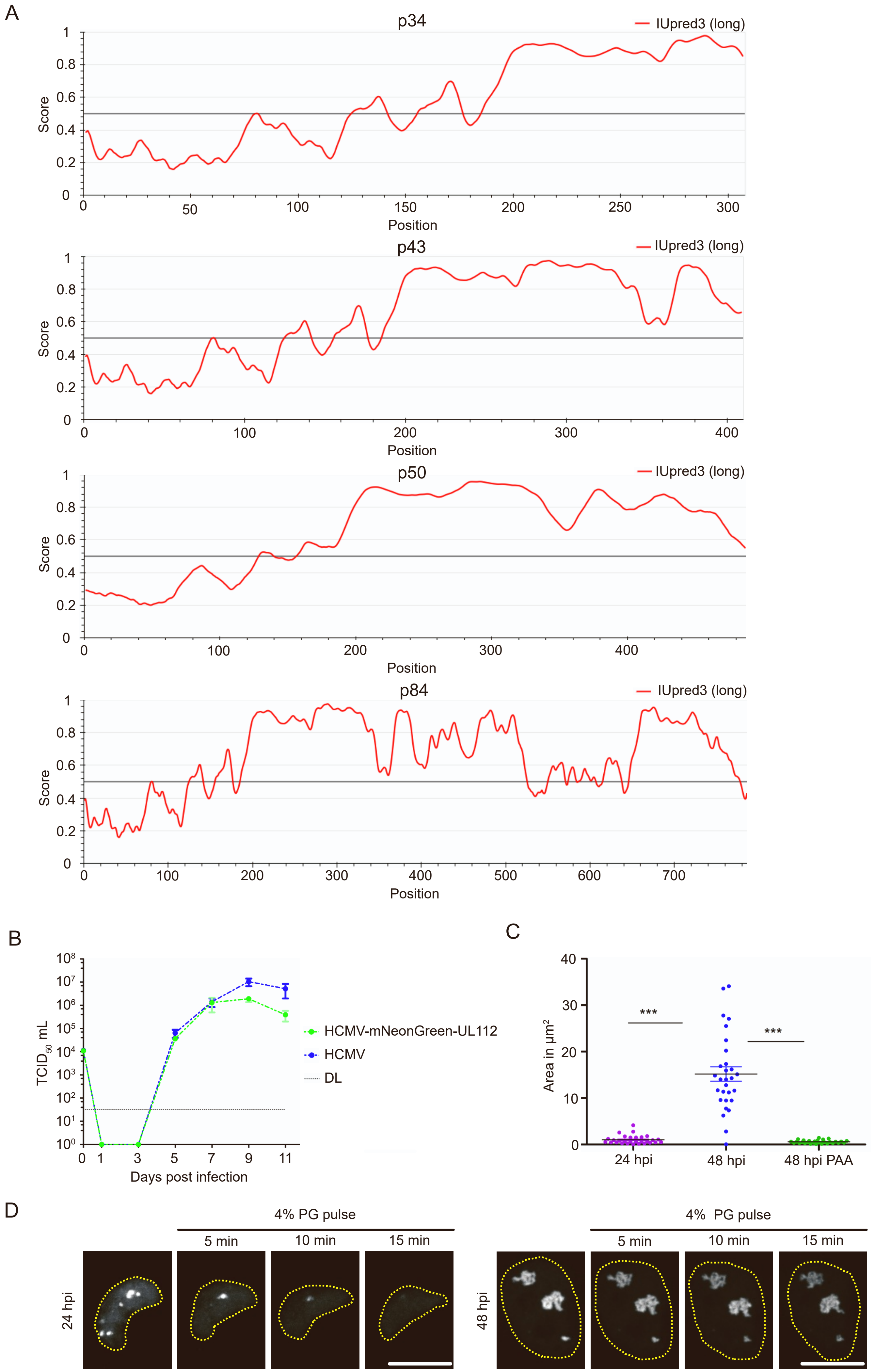
A
p34
IUpred3 (long)
Score
Position
p43
IUpred3 (long)
Score
Position
p50
IUpred3 (long)
Score
Position
p84
IUpred3 (long)
Score
Position
B
TCID50 mL
Days post infection
HCMV-mNeonGreen-UL112
HCMV
DL
C
Area in μm²
***
***
24 hpi
48 hpi
48 hpi PAA
D
4% PG pulse
5 min
10 min
15 min
24 hpi
4% PG pulse
5 min
10 min
15 min
48 hpi

Supplemental Figure 1, related to Figure 1.

(A) Disorder plots of the UL112-113 isoforms related to Fig 1. UL112-113 protein isoforms were analyzed by IUPred3 (Dosztányi et al., 2005) to predict disordered regions. The degree of disorder is scored on a scale from 0 to 1, with 1 indicating the highest level of disorder. Extended disordered regions are found in all four isoforms. The shared N-terminus is highly ordered.

(B) Replication kinetics of HCMV-mNeonGreen-UL112 and the parental HCMV strain. Cells were infected at an MOI of 0.1. Virus released into the supernatant was titrated.

(C) Quantification of the area of UL112-113 foci at 24 and 48 hpi or at 48 hpi in the presence of 250 µg/µl PAA.

(D) Treatment of infected cells with the LLPS inhibitor propylene glycol (PG, 4% w/v). MRC-5 cells were infected with HCMV-mNeonGreen-UL112 at an MOI of 1 for either 24 h or 48 h, treated with PG and imaged by live-cell spinning disk microscopy. Pictures show z-maximum intensity projection of 9 slices. The data shown are representative of three biological replicates (B) or three independent experiments (C-D). Yellow dotted lines indicate nuclear boundaries. Representative cells are shown in all panels.

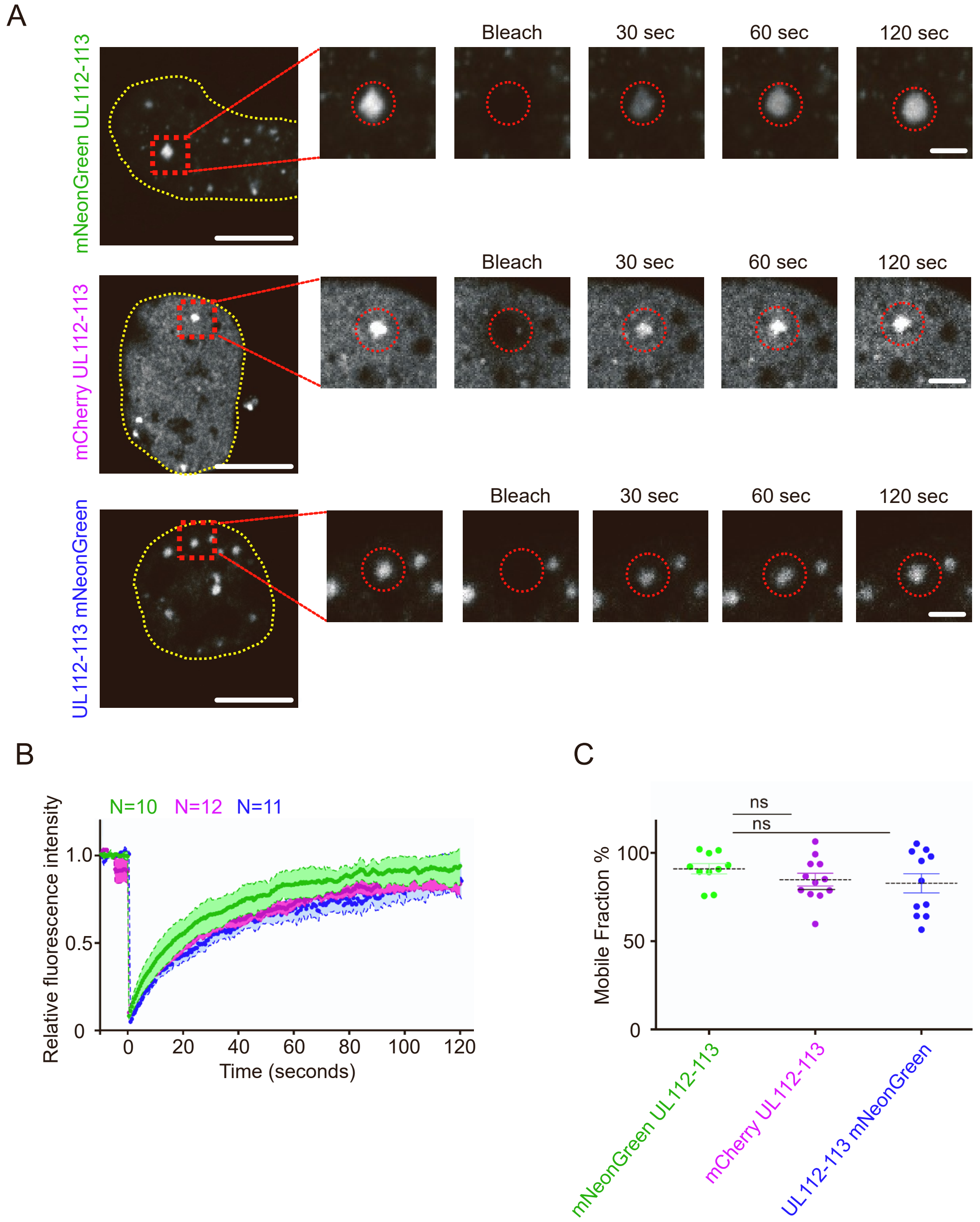

Supplemental Figure 2, related to Figure 2.

(A) Effect of tag and tag position on UL112-113 LLPS. HEK-293A cells were transfected with plasmids encoding mNeonGreen-UL112-113 (green), mCherry-UL112-113 (Magenta) or UL112-113-mNeonGreen (Blue). Cells were analyzed between 24 and 48 h post transfection. Scale bars indicate 10 µm and 2 µm in the insets.

(B) FRAP curves of transfected cells as described in (A). Curves represent an average of N different cells from 2 independent experiments.

(C) Mobile fractions from the cells analyzed in (B).

Yellow dotted lines indicate nuclear boundaries.

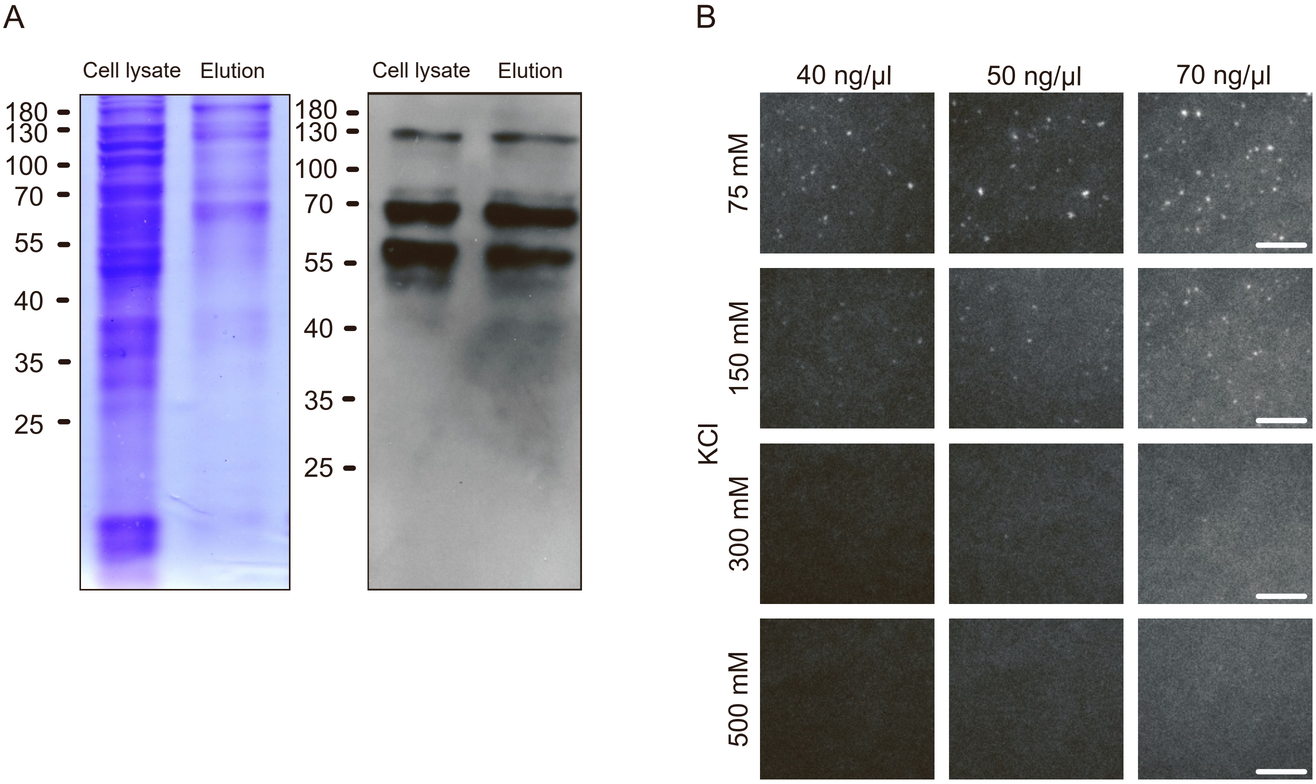

Supplemental Figure 3, related to Figure 3.

(A) Purification of UL112-113. mNeonGreen-UL112-113 expression was induced in T-REx-293 cells with 2 µg/ml Dox, and mNeonGreen-UL112-113 was purified with Ni-NTA beads. Proteins were separated on a 12% polyacrylamide gel. Protein purity was evaluated by Coomassie staining and western blot analysis. The four UL112-113 isoforms were detected with an mNeonGreen-specific antibody in the lysate (Cell lysate) and after elution from the beads (Elution).

(B) In vitro LLPS assay. A range of mNeonGreen-UL112-113 concentrations were mixed with a range of KCl buffers as indicated. Droplet formation was studied by widefield fluorescence microscopy.

(A-B) Data shown are representative of three independent experiments. Representative images are shown. Scale bar, 10 µm.

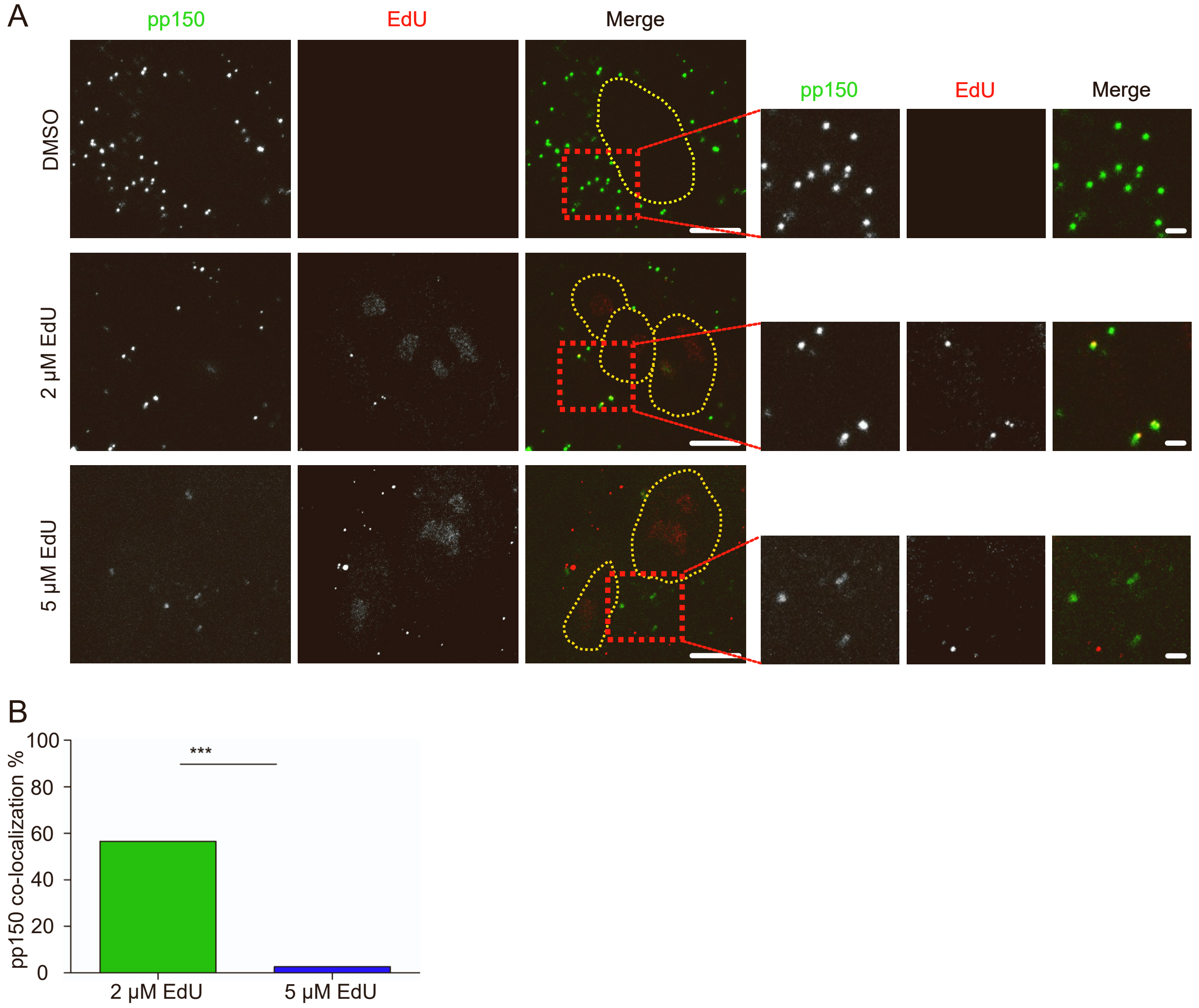

Supplemental Figure 4, related to Figure 5

(A) Association of capsids with viral genomes. HFF cells were infected (MOI 1) with HCMV-UL32-GFP that have been mock labeled (DMSO) or labeled with 2 or 5 µM EdU. UL32 encodes the capsid-associated inner tegument protein pp150. Two hpi cells were fixed and click-labeled with AF555-Picolyl-azide (EdU). Insets show EdU signals in relation to pp150-GFP labeled capsids.

(B) Quantification of genome association. Association of EdU labeled genomes with pp150-GFP capsids. The percentage capsids/ genome association is a representative example of two independent biological replicates. Yellow dotted lines indicate nuclear boundaries.

Representative images are shown. Scale bar, 10 µm.

A

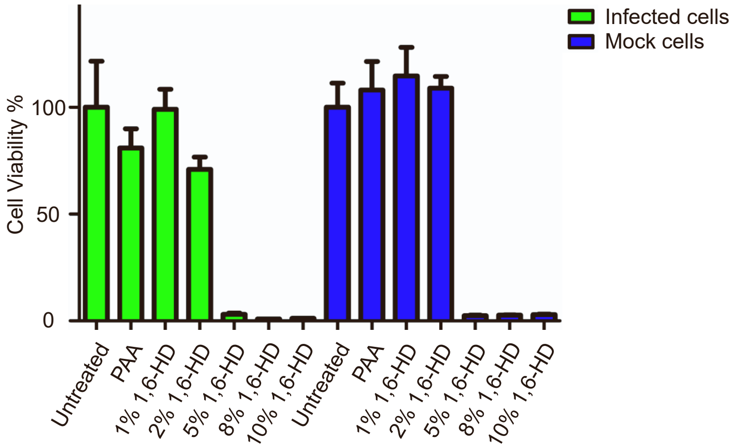

B

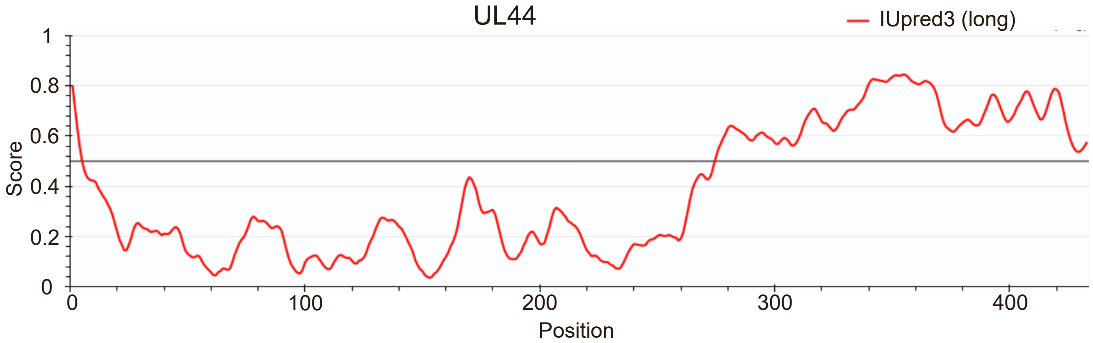

Supplemental Figure 5, related to Figure 6

(A) Cell viability after 1,6-HD treatment. Cells were infected at an MOI of 1 or mock infected. 24 hpi cells were treated with increasing amounts of 1,6-HD (1, 2, 5, 8, and 10%) or 250 µg/µl phosphonoacetic acid (PAA) for 2 hours. Cell viability was measured using an ATP assay. The percentage of cell viability is a representative example of three independent biological replicates.

(B) Disorder plot of the UL44 protein. The UL44 protein sequence was analyzed with IUPred3 (Dosztányi et al., 2005) to predict disordered regions. The degree of disorder is scored on a scale from 0 to 1, with 1 indicating the highest level of disorder. The N-terminus is predicted to be highly ordered, the C-terminus disordered.
