# Supplementary material for: Human cytomegalovirus forms phase-separated compartments at viral genomes to facilitate viral replication
Source: Cell Rep. 2022 Mar 8;38(10):110469. doi: 10.1016/j.celrep.2022.110469 (PMC8924372; doi:10.1016/j.celrep.2022.110469)
Supplement: Document S2. Article plus supplemental information [file mmc5.pdf]

Cell Reports

Article

# Human cytomegalovirus forms phase-separated compartments at viral genomes to facilitate viral replication

## Graphical abstract

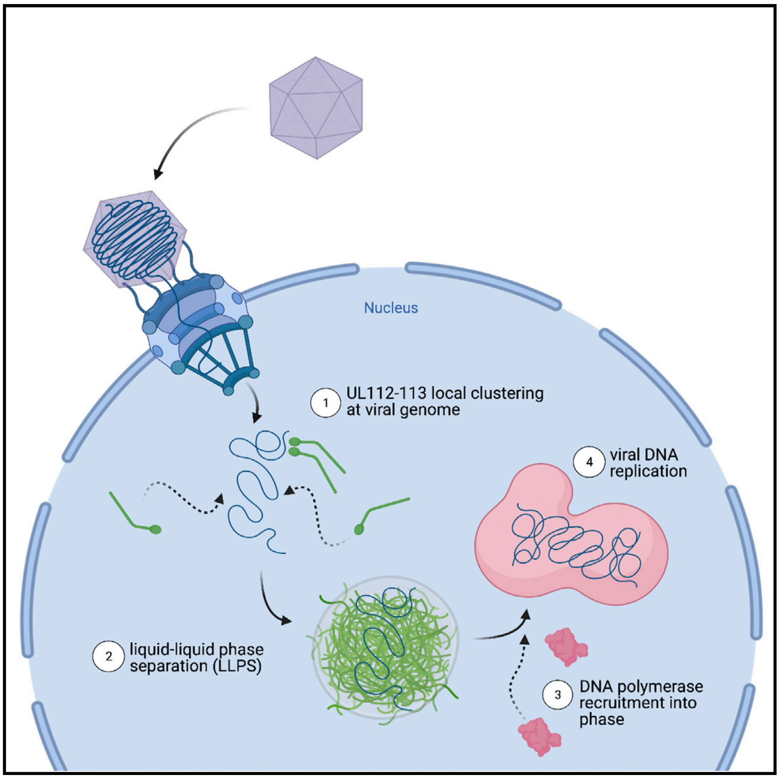


## Highlights

- HCMV pre-replication compartments are fluid biomolecular condensates
- The viral UL112-113 proteins mediate LLPS
- Incoming viral genomes nucleate phase separation
- Phase separation facilitates recruitment of replication factors

## Authors

Enrico Caragliano, Stefano Bonazza, Giada Frascaroli, ..., Kay Grünewald, Jens B. Bosse, Wolfram Brune

## Correspondence

jens.bosse@cssb-hamburg.de (J.B.B.),
wolfram.brune@leibniz-hpi.de (W.B.)

## In brief

Human cytomegalovirus replicates its DNA genome in specialized replication compartments in the host cell nucleus. Caragliano et al. show how the viral UL112-113 proteins undergo liquid-liquid phase separation at viral genomes to form replication compartments and how phase separation promotes the recruitment of the viral DNA polymerase.

Check for updates

Caragliano et al., 2022, Cell Reports *38*, 110469
March 8, 2022 © 2022 The Author(s).
https://doi.org/10.1016/j.celrep.2022.110469

CellPress

Cell Reports

CellPress
OPEN ACCESS

Article

# Human cytomegalovirus forms phase-separated compartments at viral genomes to facilitate viral replication

Enrico Caragliano,[1,2,4] Stefano Bonazza,[1] Giada Frascaroli,[1] Jiajia Tang,[1,7] Timothy K. Soh,[1,2,4,5] Kay Grünewald,[1,2,3,5] Jens B. Bosse,[1,2,4,5,*] and Wolfram Brune[1,6,8,*]

[1]Leibniz Institute for Experimental Virology (HPI), 20251 Hamburg, Germany
[2]Centre for Structural Systems Biology, 22607 Hamburg, Germany
[3]Institute of Biochemistry and Molecular Biology, University of Hamburg, 20146 Hamburg, Germany
[4]Institute of Virology, Hannover Medical School, 30625 Hannover, Germany
[5]Cluster of Excellence RESIST (EXC 2155), Hannover Medical School, Hannover, Germany
[6]German Center for Infection Research (DZIF), Partner Site Hamburg-Lübeck-Borstel-Riems, Hamburg, Germany
[7]Present address: Center for Single-Cell Omics, Shanghai Jiao Tong University School of Medicine, Shanghai 200025, China
[8]Lead contact
*Correspondence: jens.bosse@cssb-hamburg.de (J.B.B.), wolfram.brune@leibniz-hpi.de (W.B.)
https://doi.org/10.1016/j.celrep.2022.110469

## SUMMARY

**Human cytomegalovirus (HCMV) replicates its DNA genome in specialized replication compartments (RCs) in the host cell nucleus. These membrane-less organelles originate as spherical structures and grow in size over time. However, the mechanism of RC biogenesis has remained understudied. Using live-cell imaging and photo-oligomerization, we show that a central component of RCs, the UL112-113 proteins, undergo liquid-liquid phase separation (LLPS) to form RCs in the nucleus. We show that the self-interacting domain and large intrinsically disordered regions of UL112-113 are required for LLPS. Importantly, viral DNA induces local clustering of these proteins and lowers the threshold for phase separation. The formation of phase-separated compartments around viral genomes is necessary to recruit the viral DNA polymerase for viral genome replication. Thus, HCMV uses its UL112-113 proteins to generate RCs around viral genomes by LLPS to ensure the formation of a pro-replicative environment.**

## INTRODUCTION

Compartmentalization is a key feature of all living entities. A common separation mechanism in cell biology is cellular membranes that divide cells into distinct compartments. In addition, eukaryotic cells also harbor membrane-less compartments, such as P-bodies and stress granules in the cytosol and nucleoli, nuclear speckles, and Cajal bodies in the nucleus (Brangwynne et al., 2009; Fei et al., 2017; Feric et al., 2016; Fox et al., 2018; Strom and Brangwynne, 2019), which are now commonly referred to as biomolecular condensates (Banani et al., 2017). Some of these compartments have liquid properties as reflected by their round shape and their ability to fuse and relax into larger spherical structures as a result of their surface tension as well as rapid diffusion of molecules within the body (Brangwynne et al., 2009; Hyman et al., 2014; McSwiggen et al., 2019a; Patel et al., 2015). These compartments form through a process called liquid-liquid phase separation (LLPS), similar to the formation of oil droplets in water (Hyman et al., 2014). LLPS is a process in which the interactive forces between groups of molecules outweigh the surrounding solvent's forces (Alberti et al., 2019). The resulting condensed liquid droplets constitute membrane-less compartments that concentrate specific factors. Compartment formation by LLPS can increase reaction specificity and kinetics and exclude inhibitory factors. It also plays a role in buffering substrate concentrations to facilitate and promote biological reactions.

Phase-separating proteins often contain a multivalent interaction domain and an intrinsically disordered region (IDR), which drives phase separation by homotypic interactions (Hyman et al., 2014; Lin et al., 2015; Saar et al., 2021). In a binary system consisting of a phase-separating protein P and a solute S, phase separation is governed by the concentration of P in S and thermodynamic parameters, such as temperature and pH. However, heterotypic interactions with cellular polymers, such as nucleic acids or multivalent proteins (Banani et al., 2017; Shevtsov and Dundr, 2011; Shin et al., 2018), can act as local triggers that nucleate clustering and induce LLPS at specific sites below the global concentration threshold for LLPS (Bracha et al., 2018).

Since phase-separated cellular compartments, such as nucleoli and nuclear speckles, play essential roles in RNA processing, rRNA synthesis, ribosome assembly, and mRNA splicing (Feric et al., 2016; Riback et al., 2020), it is not surprising that several RNA viruses use LLPS to generate compartments for viral RNA replication or virion assembly in the cytoplasm (Alenquer et al., 2019; Cubuk et al., 2021; Geiger et al., 2021;


This is an open access article under the CC BY license (http://creativecommons.org/licenses/by/4.0/).

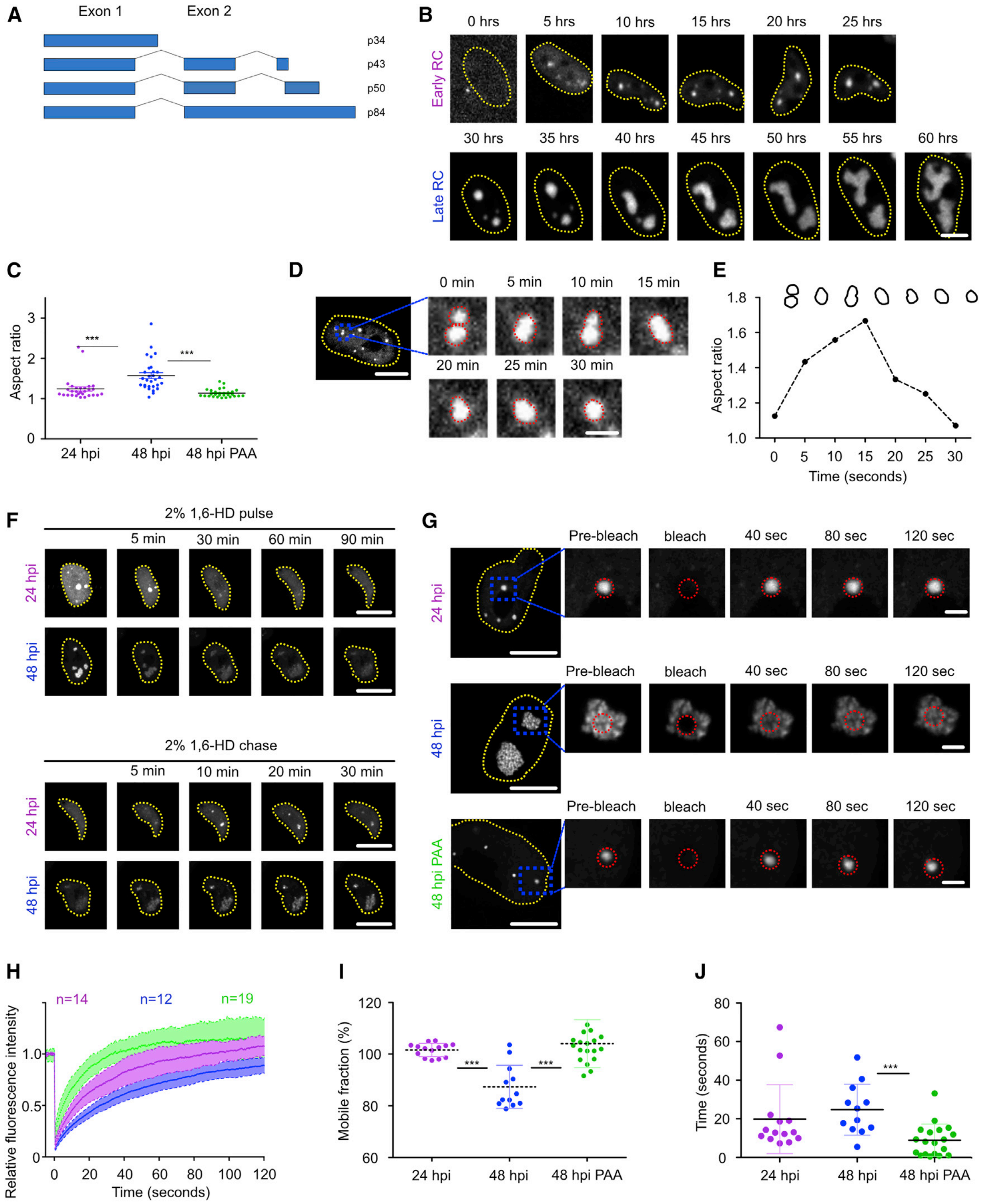


**Figure 1. Pre-replication compartments are fluid biomolecular condensates formed by LLPS**
(A) Schematic of the UL112-113 gene and its four protein products.
(B) Time-lapse images of MRC-5 cells infected with HCMV-mNeonGreen-UL112 (MOI = 10).
(C) Aspect ratios of PRCs and RCs at 24 and 48 hpi ± PAA treatment for 48 h. An aspect ratio (width:height) close to 1 indicates roundness. n = 30 per condition.
(D) PRCs fusing in MRC-5 cells infected with HCMV-mNeonGreen-UL112 (MOI = 1) at 24 hpi.
(E) Aspect ratios of fusing mNeonGreen-UL112-113 foci over time from (D).
(F) MRC-5 cells were infected with HCMV-mNeonGreen-UL112 (MOI = 1) for 24 or 48 h and treated with 2% 1,6-HD for 90 min (pulse) and imaged by spinning disk microscopy. The medium was replaced and cells were imaged for another 30 min (chase).
(G) MRC-5 cells were infected with HCMV-mNeonGreen-UL112 (MOI = 1) for 24 h, 48 h, or treated with PAA for 48 h and analyzed by FRAP.
(H) FRAP curves show means ± SD of n cells.
(I) Mobile fraction calculated from the data in (H).

(legend continued on next page)

Guseva et al., 2020; Heinrich et al., 2018; Nikolic et al., 2017; Zhou et al., 2019b). By contrast, the mechanism of RC formation employed by DNA viruses and the role of LLPS in this process remain poorly understood.

The *Herpesviridae* are a family of large double-stranded DNA viruses that replicate their genomes in the host cell nucleus. The family is subdivided into α-, β-, and γ-herpesviruses and comprises important human pathogens, such as herpes simplex virus, varicella-zoster virus, Epstein-Barr virus, and human cytomegalovirus (HCMV) (Pellet et al., 2007). HCMV, the prototype of the *Betaherpesvirinae*, is a leading cause of morbidity and mortality in immunocompromised transplant patients and is also the most frequent cause of congenital infections worldwide (Griffiths et al., 2015).

Upon HCMV infection, the viral genome is translocated to the nucleus. One of the first-expressed viral gene products is the immediate-early 1 (IE1) protein. It interacts with the promyelocytic leukemia (PML) protein and disrupts PML nuclear bodies to counteract the repressive function of these nuclear structures on viral gene transcription (Scherer and Stamminger, 2016). Viral transcription and DNA replication take place in distinct nuclear areas called viral replication compartments (RCs). Viral genome replication initiates at the origin of lytic replication (Anders and Punturieri, 1991) and requires six core replication proteins that are highly conserved among the *Herpesviridae* (Boehmer and Nimonkar, 2003): the viral DNA polymerase and its processivity factor (encoded by ORFs UL54 and UL44, respectively), the single-stranded DNA-binding protein (UL57), and the tripartite helicase-primase complex (UL70, UL102, and UL105). In addition, HCMV DNA replication depends on a few additional viral proteins, three of which are also RC components: the IE2 protein and the proteins encoded by UL84 and UL112-113 (Pari and Anders, 1993). While the function of IE2 as the major viral transactivator is well understood (Li et al., 2020; Sarisky and Hayward, 1996), much less is known about the roles of UL84 and UL112-113. UL84 localizes to the periphery of RCs (Bender et al., 2014) and is essential for replication of some HCMV strains but not for others (Sarisky and Hayward, 1996; Spector, 2015), whereas the UL112-113 locus is essential for all HCMV strains analyzed so far (Dunn et al., 2003; Kim et al., 2015; Schommartz et al., 2017; Yu et al., 2003). The UL112-113 gene gives rise to four alternatively spliced transcripts coding for four phosphoproteins (p34, p43, p50, and p84; Figure 1A) with a shared N terminus encoded by exon 1 (Schommartz et al., 2017; Wright et al., 1988). The N-terminal domain mediates interactions between the four UL112-113 isoforms. The UL112-113 proteins also interact with the DNA polymerase processivity factor UL44 (Kim and Ahn, 2010; Kim et al., 2015; Park et al., 2006) and bind to DNA (Iwayama et al., 1994). When expressed outside of the viral context, the UL112-113 proteins form intranuclear foci reminiscent of viral pre-replication compartments (PRCs) (Ahn et al., 1999; Schommartz et al., 2017). However, how the UL112-113 proteins contribute to RC formation has remained unclear.

Here, we show that LLPS induced by the HCMV UL112-113 proteins is essential for viral replication. The UL112-113 proteins consist of an interaction domain and a large IDR and form spherical compartments in the nucleus with all key properties of liquid droplets. We further show that viral DNA nucleates UL112-113 droplets, thereby ensuring that UL112-113 compartments form around genomes and establish a pro-replicative environment at a time of infection when overall UL112-113 concentrations are still low. Accumulation of the viral DNA polymerase processivity factor UL44 in UL112-113 compartments required the UL44 C-terminal IDR, and dissolution of PRCs by 1,6-hexanediol (1,6-HD) resulted in a failure to synthesize progeny viral DNA. Our results indicate that phase separation is essential for the structural integrity of PRCs and viral DNA replication. Thus, the UL112-113 proteins serve an essential function by creating a pro-replicative environment around viral genomes.

## RESULTS

### PRCs are fluid biomolecular condensates formed by LLPS

Pleomorphic HCMV nuclear RCs arise from droplet-like PRCs (Ahn et al., 1999; Iwayama et al., 1994; Penfold and Mocarski, 1997) that phenotypically resemble fluid biomolecular condensates formed by LLPS (Brangwynne et al., 2009; Hyman et al., 2014; McSwiggen et al., 2019a). To identify proteins that could play a role in HCMV PRC formation by LLPS, we performed an *in silico* analysis of the viral proteins required for viral replication (UL54, UL44, UL57, UL70, UL102, UL105, IE2, UL84, and UL112-113), investigating their content of IDRs. The UL112-113 proteins caught our attention as they are essential for viral replication and early constituents of PRCs (Ahn et al., 1999). In addition to the common ordered N terminus responsible for self-interaction (Kim et al., 2015; Park et al., 2006), the four protein isoforms encoded by the ORF UL112-113 are predicted to have highly disordered C termini containing G/S repeats of varying lengths (Figure S1A). Thus, the UL112-113 proteins may fulfill the classic prerequisites for LLPS (Bracha et al., 2018; Feric et al., 2016).

To investigate the biophysical properties of UL112-113 during HCMV infection, we constructed a recombinant virus carrying the green fluorescent protein mNeonGreen fused to the 5′ end of the UL112-113 gene. HCMV-mNeonGreen-UL112 showed only a minor growth impairment very late in infection (Figure S1B). As early as 5 h post-infection (hpi), we found strongly fluorescent pre-replication foci (PRCs) in the nuclei of infected cells (Figure 1B; Video S1). They were almost perfectly round, as indicated by their aspect ratio (Figure 1C). Importantly, we measured only foci that were too large to be diffraction limited, such that the measured sphericity was not an artifact of blurring signals by the point spread function of the microscope. In live-cell imaging, PRCs were mobile and fused over minutes, relaxing into larger spheres with aspect ratios close to one, indicating that they were fluid biomolecular condensates or droplets (Figures 1D

(J) Half-time recovery calculated from the data in (H).

The data shown are representative of three independent experiments (B, C, F–I) or three individual replicates (D and E). Yellow dotted lines indicate nuclear boundaries. Red dotted lines indicate areas of analysis. Scale bars, 10 μm and 2 μm (insets). *** $p < 0.001$.

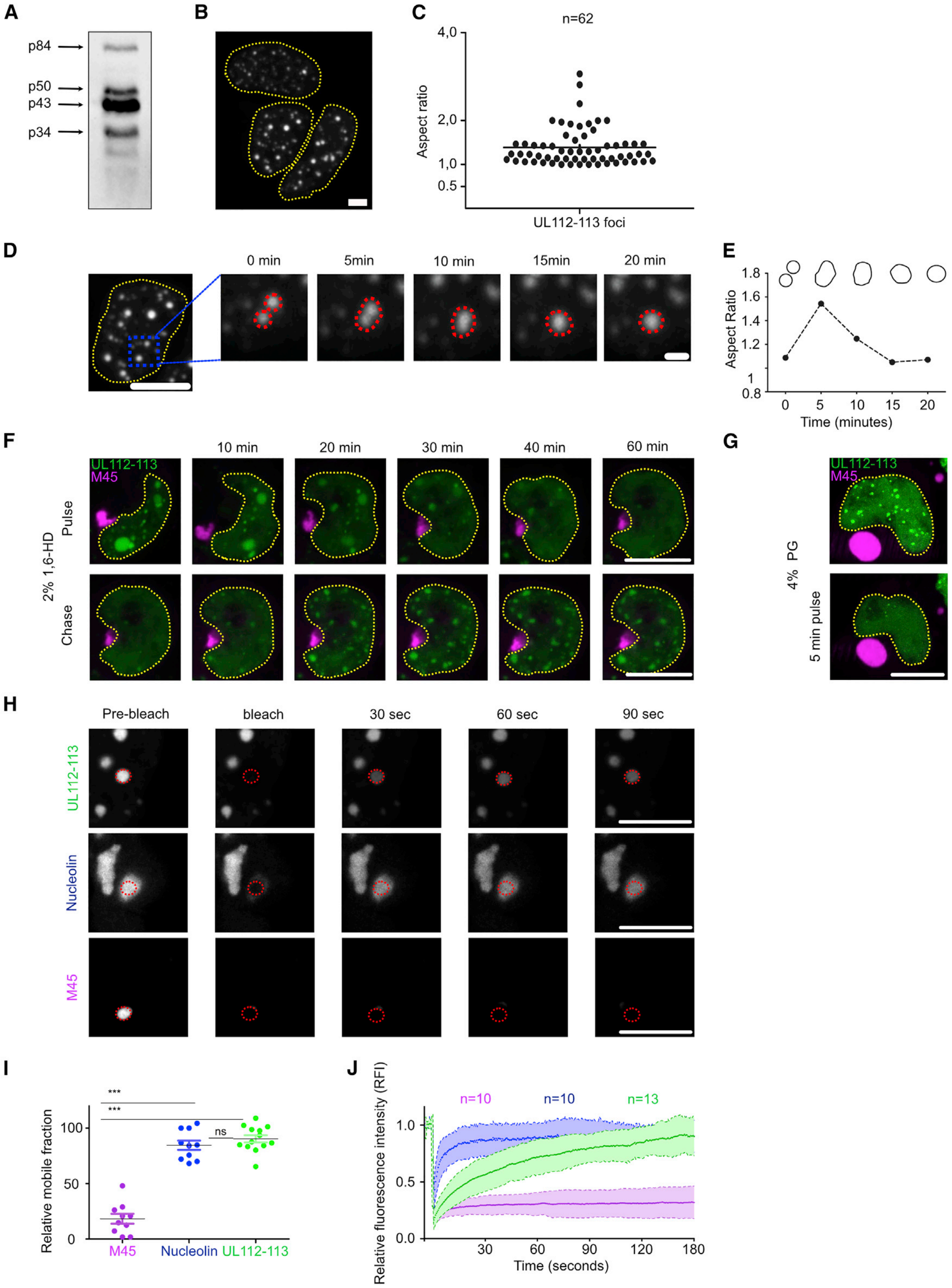


**Figure 2. UL112-113 forms liquid compartments in transfected cells**

(A) HEK293A cells were transfected with a plasmid expressing mNeonGreen-UL112-113. The four UL112-113 isoforms were detected with an anti-mNeonGreen antibody.

(B) Maximum intensity projections of cells transfected as in (A), fixed after 48 h, and analyzed by cLSM.

(C) Aspect ratios (width:height) of UL112-113 foci from (B).

*(legend continued on next page)*

and 1E). PRCs grew over time about 15-fold (Figure S1C) and transformed into pleomorphic RCs, as indicated by an increase in aspect ratios (Figures 1B and 1C; Video S1). The aliphatic alcohol 1,6-HD is commonly used as a tool to characterize biomolecular condensates (Kroschwald et al., 2015). It inhibits weak interactions leading to the dissolution of compartments formed by LLPS. We analyzed PRCs by live-cell imaging at 24 hpi and RCs at 48 hpi for their sensitivity toward 1,6-HD. Treatment with 2% 1,6-HD led to the complete dissolution of PRCs in a time frame ranging from a few minutes to 2 h. PRCs reappeared after removal of the inhibitor, indicating reversibility. By contrast, RCs at 48 hpi did not dissolve completely even after extended treatment, arguing for a more complex structure (Figure 1F; Video S2). Recently, propylene glycol (PG) has been used to dissolve liquid compartments in living cells (Geiger et al., 2021). Unlike 1,6-HD, which can be detrimental to cell viability (Kroschwald et al., 2017), PG is known to be well tolerated by cultured cells at concentrations below 5% (Mochida and Gomyoda, 1987). When we treated infected cells with 4% PG, we obtained results similar to those obtained with 1,6-HD (Figure S1D).

Next, we used fluorescent recovery after photobleaching (FRAP) to compare the dynamic recovery rates of PRCs and RCs. We observed a rapid and almost complete recovery after photobleaching of PRCs (Figures 1G and 1H). By contrast, RCs appeared to recover only partially and slower (Figures 1G and 1H) as the fraction of mobile UL112-113 molecules that could be exchanged with fluorescent ones (mobile fraction) decreased (Figure 1I). At the same time, the recovery half-time increased slightly from 24 to 48 hpi (Figure 1J). These results suggested a change in biophysical properties of PRCs that correlated with the onset of viral DNA replication right around 24 hpi (McVoy and Adler, 1994). To test if viral DNA replication is responsible for increasing the fraction of immobilized UL112-113 and the decreased diffusivity, we used phosphonoacetic acid (PAA), an inhibitor of viral DNA replication and late gene expression. In the presence of PAA, PRCs did not transform into RCs but remained small, spherical, and fluid (Figures S1C, 1C, and 1G–1J). We concluded that PRCs are phase-separated, fluid compartments that transition into biomolecular condensates with a higher fraction of bound UL112-113 molecules (i.e., RCs) after the onset of viral DNA replication.

## UL112-113 LLPS is independent of viral factors

To determine whether UL112-113 proteins can form phase-separated droplets in the absence of other viral proteins, we transfected 293T cells with a plasmid expressing mNeonGreen-tagged UL112-113. Western blots confirmed that all four isoforms were expressed (Figure 2A). Transfection resulted in numerous non-diffraction-limited, spherical structures per nucleus (Figures 2B and 2C), providing the first evidence that these structures were liquid as surface tension would induce sphericity. Time-lapse imaging data supported this conclusion as the fusion of UL112-113 foci and relaxation into larger spherical structures were regularly observed (Figures 2D and 2E), indicating surface tension. The relaxation of droplets occurred in a time frame of minutes, similarly to other low-viscosity biomolecular condensates (Brangwynne et al., 2009; Fei et al., 2017). We then tested UL112-113 droplets for sensitivity toward 1,6-HD. As a negative control, we co-transfected cells with a plasmid encoding mCherry-tagged M45, a viral protein forming insoluble cytoplasmic aggregates (Muscolino et al., 2020, 2021). Upon adding 1,6-HD to the medium, M45 aggregates were barely affected (Figure 2F), while UL112-113 droplets were rapidly dissolved (Video S3). Droplet dissolution was reversible as UL112-113 droplets reappeared after removal of 1,6-HD (Figures 2F and 2G). Similarly, treatment with 4% PG induced a fast dissolution of UL112-113 droplets but did not affect M45 cytoplasmic aggregate (Figure 2G). Based on these results, we concluded that UL112-113 undergoes LLPS and forms droplets without other viral proteins. To characterize the properties of UL112-113 droplets, we used FRAP and included fluorescently labeled Nucleolin and M45 as positive and negative controls, respectively. While UL112-113 and Nucleolin recovered almost entirely, as indicated by mobile fractions close to 100% (Figures 2H–2J), M45 recovered to less than 25%, indicating rapid exchange in UL112-113 and Nucleolin droplets and insolubility of M45 aggregates. Of note, UL112-113 recovered rapidly but with different kinetics than Nucleolin, arguing for a binding component such as DNA. Since phase separation could be affected by the oligomerization propensities of the fluorescent tag (Uebel and Phillips, 2019), we compared diffusion dynamics of mNeonGreen-UL112-113 with mCherry-UL112-113 and UL112-113-mNeonGreen. FRAP analysis of nuclear droplets showed no significant differences, neither in the recovery rates nor in the mobile fractions (Figures S2A–S2C). These data indicate that UL112-113 phase separation is not affected by the fluorescent tag choice nor by the position of the tag.

## UL112-113 proteins undergo LLPS *in vitro*

To test if UL112-113 can undergo LLPS outside of the cellular environment, we purified mNeonGreen-tagged UL112-113 from eukaryotic cells expressing mNeonGreen-tagged UL112-113 under the control of a doxycycline (Dox)-inducible promoter (T-REx-293-mNeonGreen-UL112-113) using Ni-NTA beads. This was done to obtain all four alternatively spliced isoforms and potential post-translational modifications. By Coomassie staining, we found a strong enrichment of the

(D) Time-lapse, maximum intensity projections of cells treated as in (A) depicting droplet fusion.
(E) Aspect ratios of fusing droplets marked with a red dotted line in (D).
(F) HEK293A cells were co-transfected with mNeonGreen-UL112-113 and mCherry-M45 plasmids. Cells were imaged during treatment with 2% 1,6-HD (pulse) and after its removal (chase).
(G) Cells transfected as in (F), before and after a 5 min pulse with 2% PG.
(H) HEK293A cells were transfected with plasmids encoding mNeonGreen-UL112-113, mCherry-Nucleolin as a positive and mCherry-M45 as a negative control and analyzed 2 days post-transfection by FRAP.
(I and J) Mobile fractions and FRAP curves of cells transfected as in (H). Curves show means ± SD of n cells from two independent experiments. (A–J) The data shown are representative of three independent experiments. Scale bars, 10 μm and 2 μm (insets). ***$p < 0.001$.

mNeonGreen-UL112-113 proteins in the preparations (Figure S3A). However, spectrophotometric analysis (260/280 nm ratio 0.89) indicated the presence of contaminating nucleic acids.

We then tested the propensity of the UL112-113 proteins to undergo LLPS depending on the protein and salt (KCl) concentration (Figures 3A and S3B) *in vitro*. At 40 ng/µL, the purified UL112-113 proteins formed droplets in 75 and 150 mM KCl solution. Increasing the KCl concentration to 300 mM abolished LLPS. Increasing the protein concentration to 70 ng/µl facilitated LLPS even at 300 mM KCl. No droplets were observed with the highest salt concentration of 500 mM KCl. Overall, we observed fewer droplets as the salt concentration increased in all conditions, supporting the notion that charge-based interactions are important for controlling the nucleation of UL112-113 droplets. We observed more and bigger droplets when the protein concentration was increased (Figure 3B), as expected from a protein undergoing LLPS. To test if the observed droplet formation was dependent on weak interactions, we used 1,6-HD on the highest protein concentration tested (2 µg/µl). Indeed, 1,6-HD almost completely abrogated droplet formation (Figure 3B), confirming that LLPS of isolated UL112-113 is due to weak charge-dependent protein-protein interactions. We found no droplets in the negative control (mNeonGreen alone) (Figure 3B). Altogether, these findings indicate that purified UL112-113 proteins can undergo LLPS *in vitro*.

### Clustering of the UL112-113 IDR is sufficient for LLPS

Next, we analyzed the role of the UL112-113 N- and C-terminal domains in driving LLPS. Previous reports showed that the N-terminal domain of the UL112-113 proteins is necessary for oligomerization and the formation of nuclear foci (Kim and Ahn, 2010; Kim et al., 2015), while *in silico* predictions suggested that the C termini of the UL112-113 isoforms are IDRs (Figure S1A). This protein structure fulfills the classical prerequisites for LLPS (Bracha et al., 2018; Feric et al., 2016) in which clustering by an oligomerization domain mediates close IDR interactions and subsequent LLPS. To test whether UL112-113 LLPS follows a similar principle, we replaced the N-terminal oligomerization domain with a conditional optogenetic oligomerization system recently developed to map LLPS behavior in living cells (Bracha et al., 2018). This corelet system (Figure 3C) consists of a GFP-tagged ferritin core (FTH1), which self-assembles from 24 copies of an iLID-EGFP-FTH1 fusion protein. Upon blue light activation, up to 24 copies of an IDR-mCherry-sspB fusion can bind to the ferritin core through the interaction of iLID with sspB and lead to the induction of LLPS (Figure 3C). Our analysis focused on the two essential UL112-113 isoforms, p43 and p84 (Schommartz et al., 2017). We replaced the N-terminal amino acids (aa) 1–25, which are required for oligomerization and focus formation (Kim et al., 2015; Park et al., 2006), with the mCherry-sspB oligomerization module. We then transduced HEK293A cells expressing iLID-EGFP-FTH1 cores with lentiviruses expressing p43IDR-mCherry-sspB or p84IDR-mCherry-sspB. mCherry-sspB was used as negative control (Figure 3D). Upon blue light stimulation, we observed immediate protein clustering and successive nucleation of droplets (Figure 3E). As soon as blue light illumination was stopped, droplets dissolved. Droplet formation was not observed in cells expressing mCherry-sspB alone. These results demonstrated that the predicted UL112-113 IDR mediates LLPS by local clustering in the photoactivatable corelet system.

### Exogenous DNA stimulates UL112-113 phase separation

Our FRAP analyses revealed that DNA replication led to a marked change in RC fluidity (Figures 1G–1J). As UL112-113 binds to viral DNA (Iwayama et al., 1994), we investigated whether DNA-binding influences *de novo* droplet formation. Therefore, we analyzed droplet formation in T-REx-293 cells, in which mNeonGreen-UL112-113 is expressed in a Dox-inducible fashion, and compared it with droplet formation in MRC-5 fibroblasts infected with HCMV-mNeonGreen-UL112. Surprisingly, T-REx-mNeonGreen-UL112-113 cells did not show droplets at nuclear fluorescence levels comparable with those of infected MRC-5 fibroblasts (Figure 4A). Instead, T-REx-mNeonGreen-UL112-113 cells with droplets had almost 3-fold higher UL112-113 levels than HCMV-infected cells (Figures 4A and 4B). Since infection brings viral DNA into the nucleus, we wondered if exogenous DNA could stimulate UL112-113 LLPS by lowering the concentration threshold needed for phase separation. To exclude that viral proteins induce LLPS, we infected T-REx-mNeonGreen-UL112-113 cells with replication-incompetent HSV-1. Indeed, HSV-1 induced UL112-113 LLPS at nuclear mean fluorescence intensities comparable to those of HCMV-infected cells (Figures 4A and 4B), which were approximately 3-fold lower than in cells not transfected with exogenous DNA. To verify these results, we also transfected T-REx-mNeonGreen-UL112-113 cells with an mCherry-expressing plasmid. Again, cells formed droplets at levels comparable with HCMV-infected cells and below the level for LLPS without a DNA trigger (Figures 4A–4C). We further induced T-REx-mNeonGreen-UL112-113 cells with increasing concentrations of Dox and mapped LLPS in the presence or absence of an mCherry plasmid and visually scored the resulting phenotypes. Exogenous plasmid DNA indeed triggered UL112-113 LLPS at lower levels of Dox induction and to a much higher degree (Figures 4D and 4E). Based on the results with T-REx-mNeonGreen-UL112-113 cells, we concluded that exogenous DNA could nucleate LLPS of UL112-113. Comparisons of these cells with HCMV-infected fibroblasts have to be interpreted with caution.

### UL112-113 can undergo local LLPS below the concentration threshold for global LLPS

Local nucleation of LLPS below the global threshold for LLPS has been described (Bracha et al., 2018; Du and Chen, 2018; Shin et al., 2018; Singatulina et al., 2019; Zhou et al., 2019a). This phenomenon is due to the local capture of diffusing IDRs by a few less-motile, larger scaffold molecules (diffusive capture). We wondered if UL112-113 was capable of locally inducing LLPS. To test this, we used iLID-EGFP-FTH1-expressing cells, transfected them with p84IDR-mCherry-sspB as described above, and selected cells in which global LLPS could not be induced by full-cell irradiation with blue light (Figure 4F). In these cells, local activation with a diffraction-limited activation spot resulted in local corelet clustering at the site of activation

CellPress
OPEN ACCESS

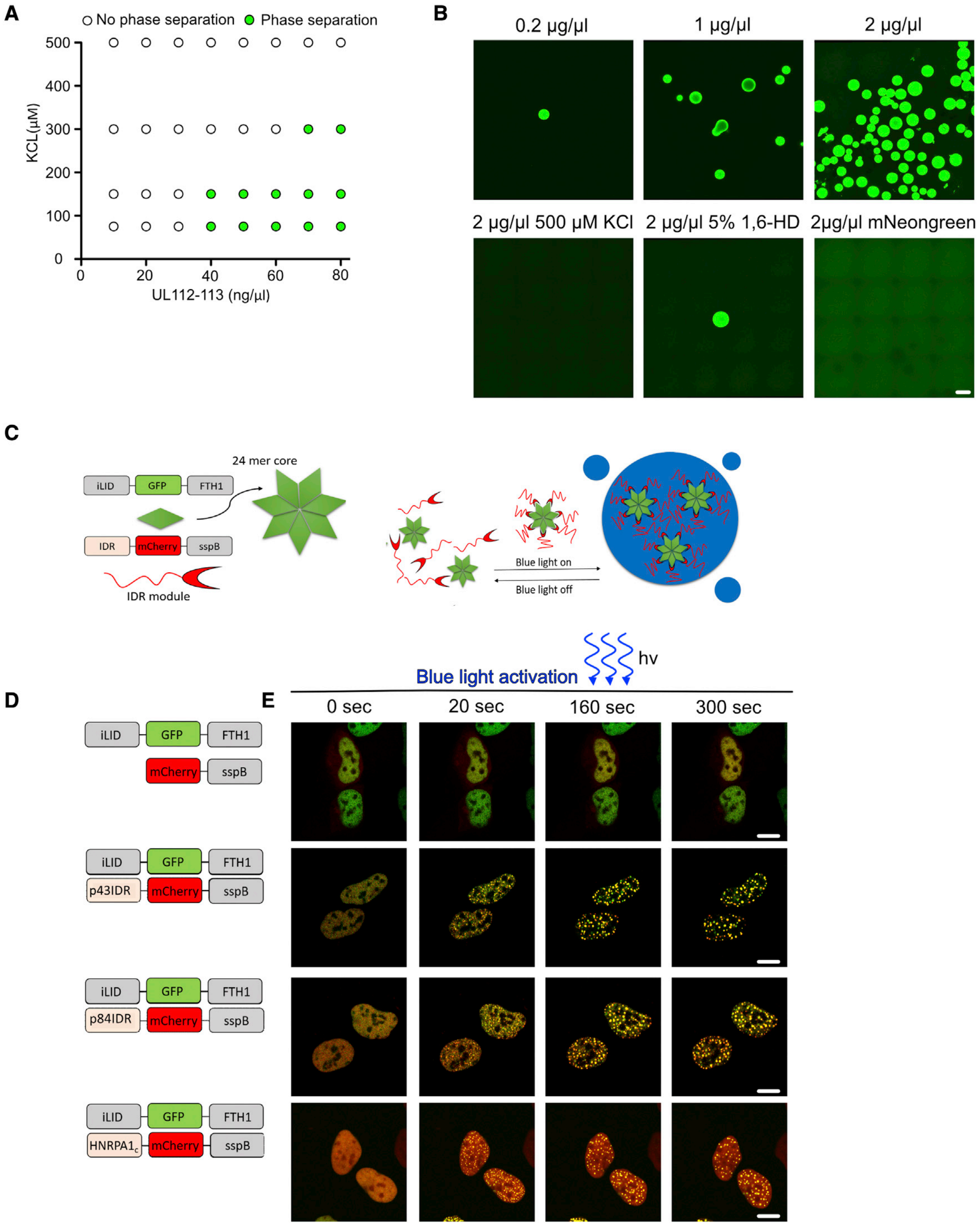


**Figure 3. Clustering of the UL112-113 IDR is sufficient for LLPS**

(A) Binary classification of phase separation *in vitro* (from Figure S3B) as a function of UL112-113 (x axis) and KCl concentrations (y axis). White dots, no phase separation; green dots, phase separation.

(B) Representative images of UL112-113 droplets. LLPS was induced by mixing purified UL112-113 proteins with a low salt (75 mM KCl) buffer. Treatment with 500 mM KCl or 5% 1,6-HD was used to inhibit phase separation. 2 μg/μL of soluble mNeonGreen was mixed with a low salt buffer. Scale bar, 100 μm.

(C) Schematic illustrating the corelet system (adapted from [Bracha et al., 2018]). iLID-EGFP-ferritin forms a 24-mer corelet (green star). sspB-mCherry (red crescent), is conjugated to IDRs from different proteins. sspB fusion proteins bind to corelets upon blue light activation, leading to LLPS (blue).

(D) Schematic of constructs. mCherry-sspB was fused to the IDRs of UL112-113 p43, p84, HNRPA1c as a positive control, or left unfused as a negative control.

(E) Photoactivation of corelet-expressing cells as depicted in (D). (A–C and E) The data shown are representative of three independent experiments. Scale bar, 10 μm.

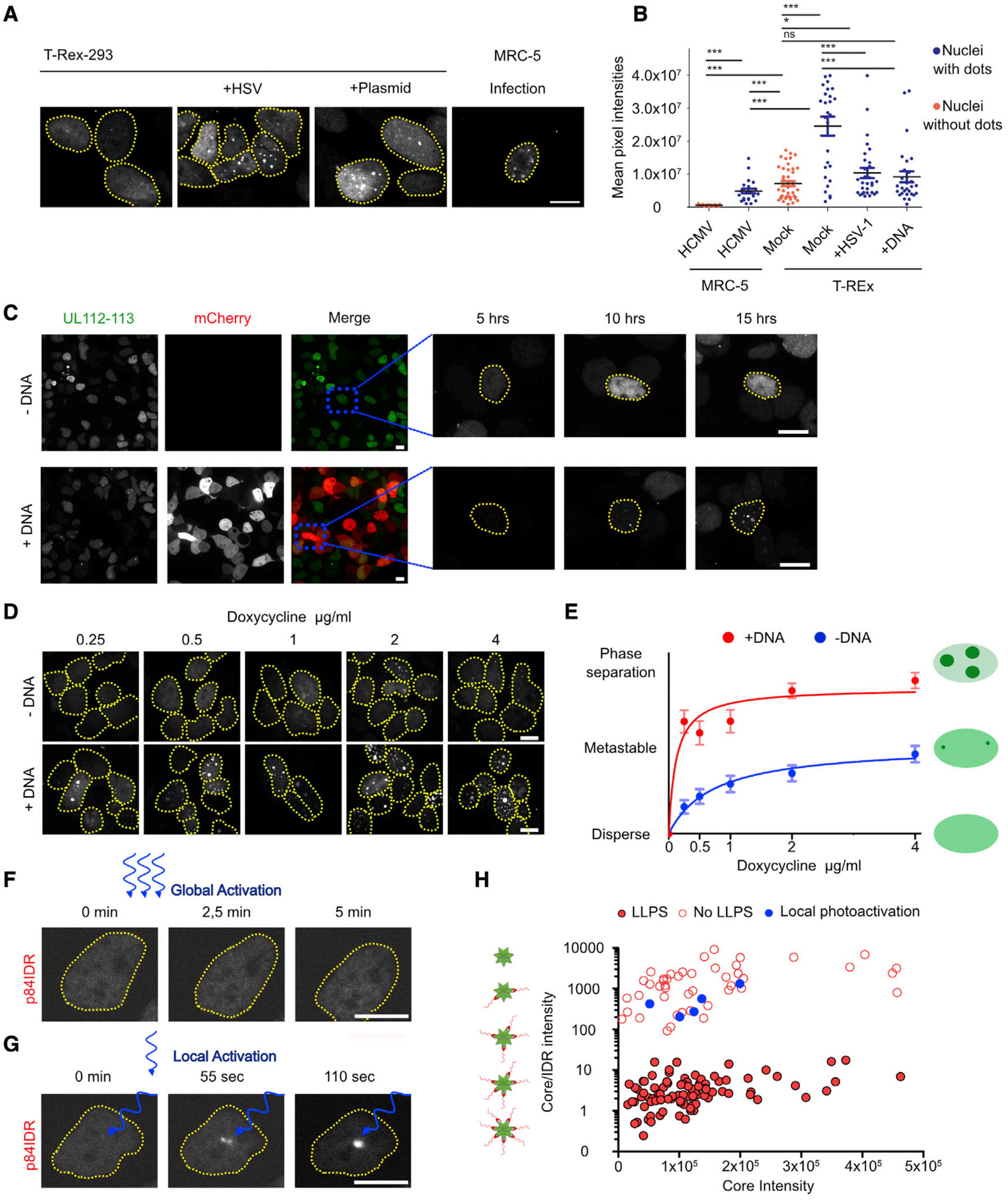


**Figure 4. Exogenous DNA stimulates UL112-113 phase separation**

(A) T-REx-293 cells expressing mNeonGreen-UL112-113 in a Dox-dependent manner were either left untreated, induced for 21 h with 2 μg/mL Dox and then infected with a replication-incompetent HSV-1 (MOI = 10) for 3 h, or transfected with plasmid DNA, incubated for 24 h, and then induced with 2 μg/mL Dox for another 24 h before fixation and imaging. MRC-5 cells infected with HCMV-mNeonGreen-UL112 (MOI = 1) for 5 h were used for comparison (MRC-5 infection). Maximum intensity projections are shown.

(B) Mean summed pixel intensities of cells in (A). Red are intensities from cells without UL112-113 dots, blue with dots.

(C) Time-lapse of UL112-113 behavior in induced T-REx cells with or without exogenous DNA. Cells were mock-transfected (−DNA) or transfected (+DNA) with an mCherry plasmid 24 h before induction with 2 μg/mL Dox for the indicated times.

(D) Maximum intensity projections of cells transfected as in (C) induced with 1–4 μg/mL Dox and imaged 24 h post-induction.

(E) Cells were manually binned into three categories. 0, disperse, no droplets; 1, metastable, some small droplets with high nuclear UL112-113 background signal; 2, phase separation, large droplets and reduced nuclear background. n = 50 cells per condition.

(F) HEK293A cells expressing iLID-EGFP-ferritin and p84IDR-mCherry-sspB globally photoactivated for 5 min.

(legend continued on next page)

CellPress
OPEN ACCESS

(Figure 4G). We also used the corelet system to test if this was due to the entrapment of diffusing IDRs by locally activated cores (Bracha et al., 2018). We mapped the phase diagram of the p84 IDR in its native cellular environment (Figure 4H). This diagram plots the relation between the core/IDR fluorescence intensity ratio to the core fluorescence intensity. It gives information on the IDR fluorescence intensities needed to drive global LLPS in relation to the intensities of cores (Figure 4H). Moreover, it describes in which density range IDRs undergo LLPS and which density the resulting phase can have. In Figure 4H, solid red dots represent individual cells in which UL112-113 could undergo LLPS after photoactivation, while empty red circles mark cells in which this did not happen. Together, the solid symbols mark the conditions under which phase separation occurs in the phase diagram, while empty symbols indicate the conditions under which LLPS does not occur. We then measured the same values in cells that did not show global LLPS before and after local activation and indicated the area in the diagram (blue circles). Consistent with local LLPS by diffusive capture, local activation allowed LLPS in an area of the diagram where global activation would not provide enough binding IDRs (Figure 4H).

## UL112-113 droplets form at viral genomes and are needed for viral genome replication

Since our data suggested that UL112-113 droplets appear at lower nuclear concentrations when exogenous DNA is introduced, and the UL112-113 p84 IDR is capable of local LLPS, we asked if viral genomes could induce local LLPS of UL112-113. To address this question, we first tested if viral genomes labeled with the nucleotide analog 5-ethynyl-2′-deoxyuridine (EdU) were incorporated into infectious viral particles. We infected MRC-5 cells with HCMV-UL32-GFP (Sampaio et al., 2005), which expressed a fluorescent, capsid-associated inner tegument protein pp150, and incubated them with different amounts of the EdU. We then purified EdU-labeled virions and used them to infect new MRC-5 cells. After fixation at 2 hpi, we used Click chemistry to label EdU-containing genomes with Alexa Fluor 555. When labeling was done with 2 $\mu$M EdU, we found most of the EdU-labeled genomes associated with pp150-GFP-labeled capsids. By contrast, we observed a much lower association when a higher EdU concentration was used (Figures S4A and S4B). We did not detect any EdU-positive particles in the unlabeled virus preparation (Figure S4A). Therefore, we decided to use 2 $\mu$M EdU for the following experiments. We produced an HCMV-mNeonGreen-UL112 EdU-labeled virus stock and infected MRC-5 cells. After fixation at 24 hpi, we found most incoming genomes colocalizing with PRCs (Figures 5A and 5B). Together with our previous findings that exogenous DNA and local clustering stimulate UL112-113 LLPS, this observation suggested that droplet nucleation is induced by incoming viral genomes.

Next, we asked whether the presence of UL112-113 is needed for genome replication. To test this, we did an experiment similar to the one above but added bromodeoxyuridine (BrdU) at 24 hpi to label newly synthesized genomes. They were detected using a BrdU-specific antibody while the incoming genomes were marked by Click-labeled EdU. At 30 hpi, we were able to distinguish replicating from non-replicating genomes by BrdU incorporation. Importantly, we found that only genomes associated with UL112-113 were amplified, as indicated by the increased area of BrdU labeling around EdU-labeled foci, suggesting that UL112-113 droplets facilitate viral genome replication (Figures 5C and 5D).

## UL112-113 LLPS promotes accumulation of the viral DNA polymerase accessory factor UL44 at viral genomes

To directly probe the role of UL112-113 phase separation at viral genomes, we labeled replicating genomes with EdU and treated infected cells 24 hpi with 1,6-HD. We carefully titered the amount and incubation time of 1,6-HD to avoid non-specific effects due to cytotoxicity (Figure S5A). RCs, as indicated by UL112-113, were undetectable after treatment with 1,6-HD for 2 h (Figures 6A and 6B). Importantly, 1,6-HD treatment resulted in an even greater reduction in EdU-positive genome areas than with the replication inhibitor PAA (Figures 6A and 6C). As UL112-113 has been described to interact with the DNA polymerase accessory factor UL44 (Kim and Ahn, 2010; Park et al., 2006), we wondered if UL112-113 LLPS is needed for UL44 accumulation at viral genomes. Indeed, dispersion of UL112-113 droplets by 1,6-HD led to complete UL44 dissociation, and no genome replication could be detected by EdU labeling (Figures 6A and 6D). To confirm that the recruitment of UL44 to PRCs involves phase separation, we analyzed its domain structure. Disorder prediction by IUPred3 indicated that UL44 consists of an ordered N-terminal part (aa 1–290) and a largely disordered C-terminal part (Figure S5B). As the C-terminal part of UL44 (aa 291–433) has been reported to be required for colocalization with UL112-113 foci and RCs (Kim and Ahn, 2010; Silva et al., 2010), we hypothesized that phase separation drives UL44 accumulation in UL112-113 droplets. If this is the case, deleting the IDR should be sufficient to abrogate colocalization with UL112-113. Indeed, we found that UL44 lacking its IDR did not accumulate in UL112-113 droplets (Figures 6E and 6F). Addition of the disordered domain (IDR) of UL112-113 isoform p43 to the UL44ΔIDR mutant rescued UL44 accumulation in UL112-113 droplets. Importantly, the C-terminal part of UL44 (aa 291–433) containing the IDR and the NLS was sufficient for colocalization with UL112-113 droplets (Figures 6E and 6F), even though the same UL44 region did not bind to UL112-113 p84 in co-immunopreciptitation experiments (Kim and Ahn, 2010). These results argue for a model in which UL44 is recruited into phase-separated PRCs through its IDR.

In summary, these data indicate that LLPS of UL112-113 is a major factor in the spatial organization of HCMV PRCs and an

(G) Cells from (F) photoactivated locally resulting in local LLPS.
(H) *In vivo* phase diagram of p84IDR-mCherry-sspB given as core fluorescence intensity and core-to-IDR ratio (Bracha et al., 2018). Solid red circles, LLPS after 10 min of photoactivation; empty red circles, no LLPS; blue circles, LLPS after local activation see (G). Data are pooled from three independent experiments. (A–F) The data shown are representative of three independent experiments. Scale bar, 10 $\mu$m. ***$p < 0.001$, *$p < 0.05$, ns not significant.

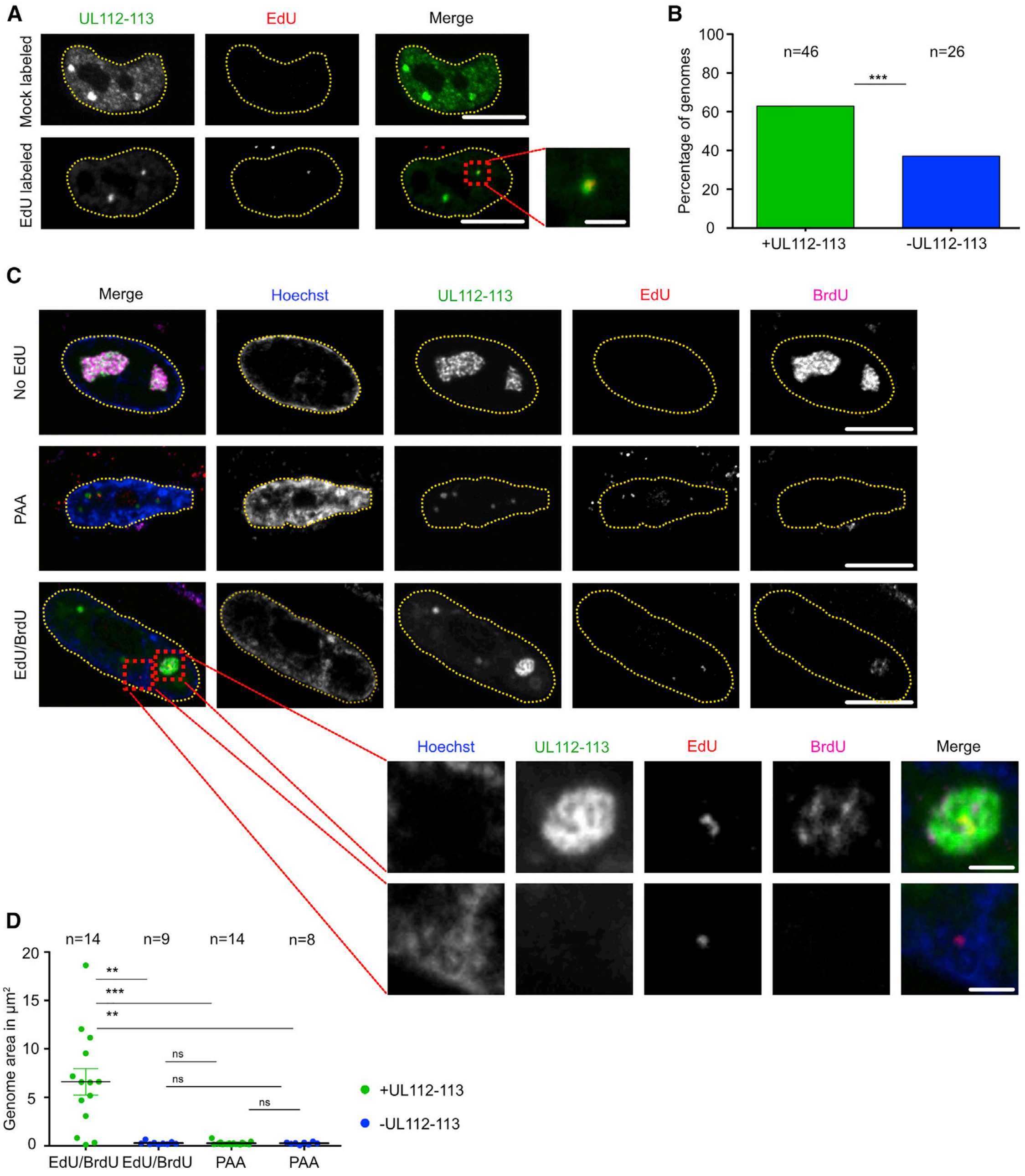


**Figure 5. UL112-113 droplets form at viral genomes and facilitate viral DNA replication**

(A) Association of UL112-113 droplets with viral genomes. HFF were infected with EdU-labeled or unlabeled HCMV-mNeonGreen-UL112 (MOI = 10). Cells (24 hpi) were fixed, Click-labeled with AF555-Picolyl-azide, and counterstained with Hoechst 33342. The inset depicts an EdU-labeled genome associated with a UL112-113 droplet.

(B) Quantification of EdU-labeled genomes associated with UL112-113 droplets. n = total number of genomes counted in 44 cells.

(C) Effect of UL112-113 droplet association on genome replication. MRC-5 cells were infected with EdU-labeled HCMV-mNeonGreen-UL112 (MOI = 1). At 24 hpi, cells were pulsed for 6 h with 100 µM BrdU and fixed. EdU-labeled incoming genomes were Click-labeled and nuclei were counterstained with Hoechst 33342. BrdU was detected by indirect immunofluorescence. Upper inset, replicating genome; lower inset, non-replicating genome.

(D) Quantification of genome area as a function of UL112-113 association. Incoming EdU viral genome replication was detected through incorporation of BrdU. EdU- and BrdU-labeled summed areas were scored as UL112-113 positive or negative. n = number of incoming EdU-labeled genomes analyzed. (A–D) The data shown are representative of three independent experiments. Scale bars, 10 µm and 2 µm (insets). ***$p < 0.001$, **$p < 0.01$, ns not significant.

CellPress
OPEN ACCESS

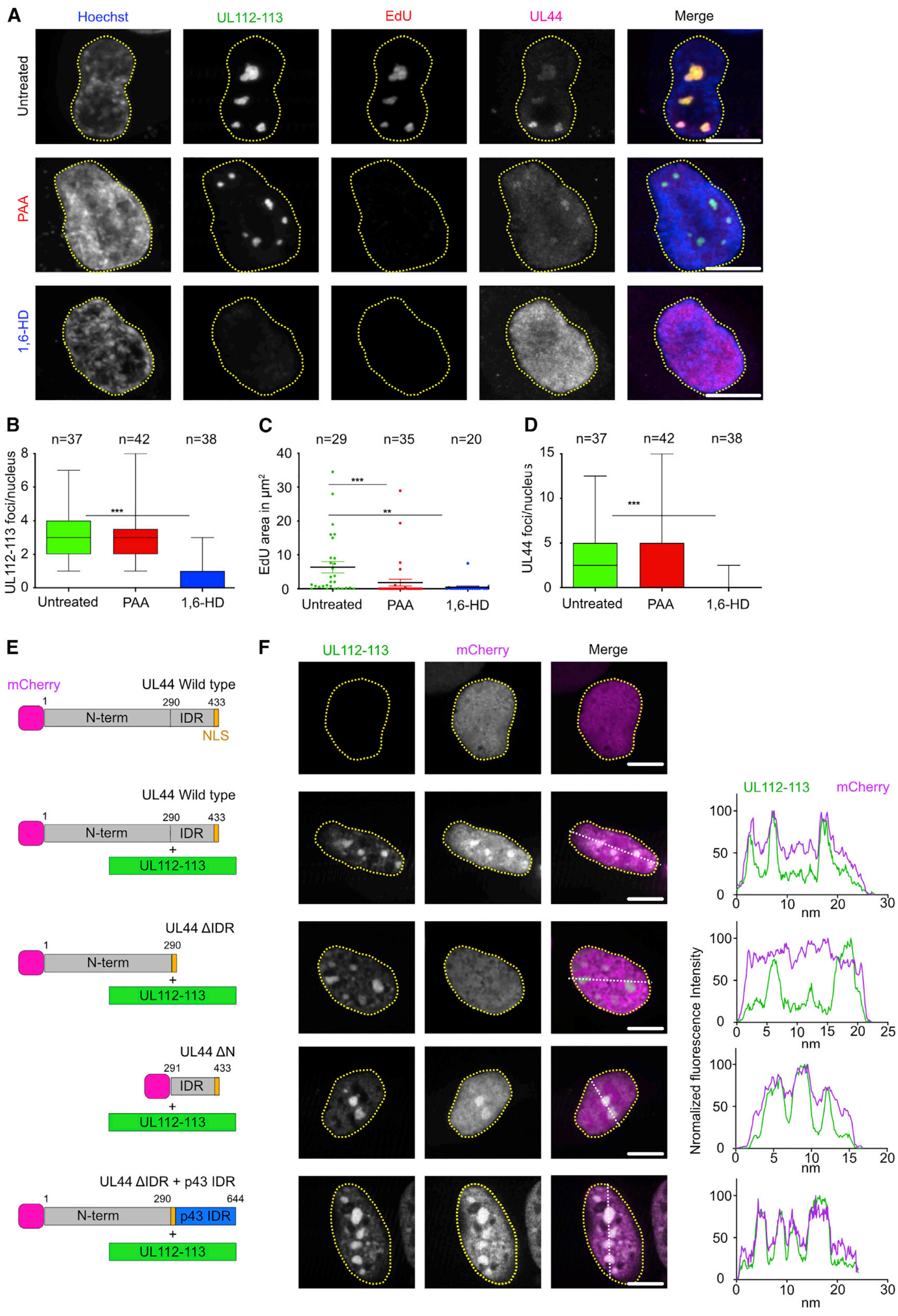


(legend on next page)

essential factor for the recruitment of proteins necessary for viral DNA replication (such as UL44) to viral genomes to facilitate their replication.

## DISCUSSION

In this study, we show that the HCMV UL112-113 proteins harbor large IDRs that mediate LLPS when clustered. They form spherical droplets in the nucleus, which can fuse and quickly regain a spherical shape, presumably due to surface tension. UL112-113 droplets are sensitive to disturbance by 1,6-HD or PG. Thus, they display properties of phase-separated compartments (Brangwynne et al., 2009; Hyman et al., 2014; McSwiggen et al., 2019a). LLPS was independent of other viral proteins and could be induced *in vitro*. Importantly, local clustering of the UL112-113 p84 IDR can induce LLPS below the global threshold, and viral DNA triggers the induction of UL112-113 droplets. Furthermore, UL112-113 droplet formation mediates recruitment of the viral polymerase accessory factor UL44 and ensures viral genome replication. Thus we conclude that HCMV uses induction of local LLPS below the global phase separation threshold to ensure UL112-113 droplet formation specifically at viral genomes, recruitment of UL44, and viral genome replication.

Cells have evolved to induce ad-hoc compartments by LLPS to dynamically react to cues, such as DNA damage, pathogen-associated molecular patterns, or to regulate gene transcription (Cho et al., 2018; Du and Chen, 2018; Patel et al., 2015). While several viral phase-separated compartments have been described for RNA and recently also for DNA viruses (Seyffert et al., 2021; Vladimirova et al., 2021), their biological function has remained largely obscure. Our data indicate that HCMV genomes serve as a polymeric scaffold for nucleation of UL112-113 by locally inducing LLPS. Triggering LLPS at genomes appears to be the mechanism by which the virus ensures that the initially limited amounts of UL112-113 early in infection are specifically concentrated as droplets at genomes. Droplet growth can be governed by a principle called Ostwald ripening, although biological systems are more complex than simple binary systems (Lee et al., 2021). Ostwald ripening describes the propensity of existing droplets to grow, while the growth of small droplets is disfavored as small droplets have a higher surface-to-volume ratio. Triggering UL112-113 LLPS locally below the global threshold for LLPS might allow the virus to ensure that UL112-113 LLPS is initiated at viral genomes. Due to Ostwald ripening, these initial droplets will preferably recruit UL112-113 and provide a pro-replicative environment while the emergence of additional droplets is disfavored. This principle might also explain why the number of replicating genomes is limited in herpesvirus-infected cells, regardless of the multiplicity of infection used (Cohen and Kobiler, 2016; Kobiler et al., 2010).

After initiation of UL112-113 droplet formation, their properties gradually change after the onset of viral DNA replication, when PRCs turn into RCs. RCs become substantially larger, acquire a less spherical and more irregular shape, and the fraction of immobile UL112-113 molecules increases. Inhibition of viral DNA replication prevents this transition, suggesting that the long concatemeric genomes generated during the rolling-circle replication process alter the liquid properties of RCs. However, it is also possible that late viral proteins, which are expressed only after viral DNA replication, contribute to this change.

HCMV PRCs and RCs consist of several viral proteins as well as proteins of cellular origin (Strang, 2015). Moreover, the UL112-113 proteins comprise four isoforms derived from differentially spliced transcripts. Currently, we do not understand how these modulate the biophysical properties of PRCs. Maybe the four isoforms allow the virus to fine-tune the formation and the biophysical properties of PRCs. The isoforms carry IDRs of different lengths, which might modulate the fluidity of UL112-113 droplets. Also, splicing might be used to regulate the expression, stability, or post-translational modification of individual isoforms. Such regulation could allow the virus to adapt to its environment, activating the lytic replication cycle preferentially in specific cell types while favoring latency or abortive infection in others.

The UL112-113 gene is conserved among the *Betaherpesvirinae*, most notably among CMVs of different animals. The best-studied homolog is the M112-113 gene of mouse CMV. It is spliced in a very similar fashion as UL112-113 and also encodes four isoforms, which form PRCs in the host cell nucleus (Ciocco-Schmitt et al., 2002; Schommartz et al., 2016). Human herpesviruses 6 and 7, two other β-herpesviruses, also encode homologs of UL112-113 (Taniguchi et al., 2000), but their function has not been studied in detail. Obvious sequence homologs of UL112-113 have not been identified in α- or γ-herpesviruses. Thus, it appears likely that the α- and γ-herpesviruses have evolved other proteins capable of forming fluid PRCs by LLPS. Interestingly, one study has shown that expression of HCMV UL112-113 by plasmid transfection stimulates the replication of KSHV, suggesting that the formation of phase-separated compartments is beneficial also for KSHV (Wells et al., 2009).

The mechanism of viral DNA replication and the formation of RCs has been studied most extensively in HSV-1, the

**Figure 6. Phase separation of UL112-113 facilitates recruitment of the viral DNA polymerase accessory factor UL44 to genomes**

(A) Influence of inhibition of UL112-113 LLPS on genome replication. MRC-5 cells were infected with HCMV-mNeonGreen-UL112 (MOI = 1). At 24 hpi, cells were pulsed for 2 h with 10 μM EdU to label newly synthesized DNA. At the same time, cells were treated with PAA (250 μg/μL), 2% 1,6-HD, or left untreated. Cells were Click-labeled with AF555-Picolyl-azide (EdU), counterstained with Hoechst 33342, and UL44 was detected by indirect immunofluorescence.

(B–D) Influence of 1,6-HD on UL112-113 foci and genome replication. Number of UL44 positive foci, area of genome labeling, and number of UL44 positive foci under conditions as in (A). Only cells expressing UL112-113 and UL44 were analyzed. n = number of foci per nucleus analyzed.

(E) Schematic of mCherry-tagged UL44 constructs. UL44 consists of an ordered N-terminal region (aa 1–290), a disordered C-terminal IDR (aa 291–424), and an NLS (aa 425–433).

(F) HEK293A cells were co-transfected with UL44 and UL112-113 plasmids as indicated in (E) and imaged 48 h post-transfection. Intensity plots illustrate signal profiles along the white dotted lines. (A–D and F) The data shown are representative of three independent experiments. ***$p < 0.001$, **$p < 0.01$.

prototype of the *Alphaherpesvirinae*. A recent study by McSwiggen and colleagues has analyzed the biophysical properties of HSV-1 RCs using the cellular RNA polymerase II (RNA Pol II) as an indirect reference molecule (McSwiggen et al., 2019b). The authors of the study showed that HSV-1 RCs share several, but not all, properties of phase-separated compartments and concluded that compartmentalization occurs through a mechanism distinct from LLPS as RNA Pol II was able to freely diffuse in and out of the compartment. By contrast, a recent study by Seyffert et al. (2021) presented evidence that the HSV-1 protein ICP4, which is an essential RC constituent, forms biomolecular condensates. However, the ICP4 condensates were more viscous than the HCMV UL112-113 droplets described here. Therefore, it is currently unclear which role compartment formation by LLPS plays in HSV-1. Comparing these studies with ours, one might conclude that RC formation differs significantly between HSV-1 and HCMV. Indeed, RC formation and viral DNA replication proceed considerably faster in HSV-1 than in HCMV. Whereas HSV-1 DNA replication commences as early as 3 hpi, it does not start until 24 hpi in HCMV (McVoy and Adler, 1994). Hence, there is probably a much greater need for HCMV to create a protected phase-separated compartment in order to prevent sequestration or silencing of the viral genome by cellular factors. However, another interpretation is also conceivable. The biophysical properties of HSV-1 RCs are somewhat similar to those of HCMV RCs at late times post-infection. However, we show that the precursors of RCs, the PRCs, possess the classical properties of phase-separated compartments. Interestingly, an HSV-1 protein mentioned in the study by McSwiggen et al. might be involved in the formation of PRCs, in a very similar manner to UL112-113 in HCMV. The HSV-1 ICP0 protein has a distinct IDR, is expressed in the earliest phase of viral infection, and forms spherical structures in the nucleus, even in the absence of other viral proteins (Everett, 1988). Similar to UL112-113, the ICP0-encoding gene is one of the very few spliced HSV-1 genes. Thus, it remains possible that the formation of phase-separated PRCs is a conserved feature of all herpesviruses.

## Limitations of the study

This study provides ample evidence for LLPS as a driving force of PRC formation at viral genomes by the HCMV UL112-113 proteins. It also shows that the UL112-113 proteins possess the typical properties of phase-separating proteins: a multivalent interaction domain and an IDR. However, previous studies have suggested that HCMV proteins associate in RCs by classical protein-protein interactions (Ahn et al., 1999; Kagele et al., 2012; Kim and Ahn, 2010; Strang, 2015). The current concept of LLPS clearly supports a role of both, direct protein-protein interactions and IDR-dependent weak interactions in the formation of liquid organelles. However, the relative contribution of the individual types of interactions have not been addressed in this study. This study also did not show how the four UL112-113 isoforms contribute to droplet formation and how the biophysical properties of PRCs change after the onset of viral DNA replication. These important questions will have to be addressed in future studies.

## STAR★METHODS

Detailed methods are provided in the online version of this paper and include the following:

- KEY RESOURCES TABLE
- RESOURCE AVAILABILITY
  - Lead contact
  - Materials availability
  - Data and code availability
- EXPERIMENTAL MODEL AND SUBJECT DETAILS
  - Cells and viruses
  - Plasmids, transfection, and transduction
- METHODS DETAILS
  - *In vitro* phase separation assay
  - Hexanediol and propylene glycol treatment
  - EdU labeling
  - Western blotting
- MICROSCOPY AND IMAGE ANALYSIS
  - Microscopy modalities
  - Fluorescence labeling
  - Fluorescence recovery after photo bleaching and photoactivation
  - Fluorescence intensity profiles
  - EdU surface area measurements for quantification of viral genome replication and colocalization
- QUANTIFICATION AND STATISTICAL ANALYSIS

## SUPPLEMENTAL INFORMATION

Supplemental information can be found online at https://doi.org/10.1016/j.celrep.2022.110469.

## ACKNOWLEDGMENTS

We thank Roland Thünauer (HPI Microscopy Facility) for imaging support, Jin-Hyun Ahn for providing plasmids, and Clifford Brangwynne and Daniel Lee for plasmids and helpful discussions. HCMV-UL32-GFP and HSV-1 in1374 were kindly provided by Lüder Wiebusch and Chris Boutell, respectively. This project was funded by the HPI Strategic Incentive Program and is part of the Leibniz Science Campus InterACt (grant agreement no. W6/2018). The HPI is supported by the Free and Hanseatic City of Hamburg and the Federal Ministry of Health. K.G. and J.B.B. are funded by the Deutsche Forschungsgemeinschaft (DFG) (German Research Foundation) under Germany's Excellence Strategy EXC 2155—project no. 390874280, by the Wellcome Trust through a Collaborative Award (209250/Z/17/Z) to K.G. and J.B.B., and through the DFG Research Unit FOR 5200 (BO 4158/5-1) to J.B.B. W.B. is funded by the German Center for Infection Research (DZIF).

## AUTHOR CONTRIBUTIONS

Conceptualization, W.B., J.B.B., and E.C.; investigation, E.C., S.B., and T.K.S.; writing – original draft, W.B., J.B.B., and E.C.; writing – review & editing, all authors; supervision, G.F., J.T., K.G., W.B., and J.B.B.; funding acquisition, K.G., J.B.B., and W.B.

## DECLARATION OF INTERESTS

The authors declare no competing interests.

Received: June 16, 2021
Revised: November 29, 2021
Accepted: February 10, 2022
Published: March 8, 2022

## REFERENCES


Ahn, J.H., Jang, W.J., and Hayward, G.S. (1999). The human cytomegalovirus IE2 and UL112-113 proteins accumulate in viral DNA replication compartments that initiate from the periphery of promyelocytic leukemia protein-associated nuclear bodies (PODs or ND10). J. Virol. *73*, 10458–10471.

Alberti, S., Gladfelter, A., and Mittag, T. (2019). Considerations and Challenges in Studying Liquid-Liquid Phase Separation and Biomolecular Condensates. Cell *176*, 419–434.

Alenquer, M., Vale-Costa, S., Etibor, T.A., Ferreira, F., Sousa, A.L., and Amorim, M.J. (2019). Influenza A virus ribonucleoproteins form liquid organelles at endoplasmic reticulum exit sites. Nat. Commun. *10*, 1629.

Anders, D.G., and Punturieri, S.M. (1991). Multicomponent origin of cytomegalovirus lytic-phase DNA replication. J. Virol. *65*, 931–937.

Banani, S.F., Lee, H.O., Hyman, A.A., and Rosen, M.K. (2017). Biomolecular condensates: organizers of cellular biochemistry. Nat. Rev. Mol. Cell Biol. *18*, 285–298.

Bender, B.J., Coen, D.M., and Strang, B.L. (2014). Dynamic and nucleolin-dependent localization of human cytomegalovirus UL84 to the periphery of viral replication compartments and nucleoli. J. Virol. *88*, 11738–11747.

Boehmer, P., and Nimonkar, A. (2003). Herpes virus replication. IUBMB Life *55*, 13–22.

Bracha, D., Walls, M.T., Wei, M.-T., Zhu, L., Kurian, M., Avalos, J.L., Toettcher, J.E., and Brangwynne, C.P. (2018). Mapping local and global liquid phase behavior in living cells using photo-oligomerizable seeds. Cell *175*, 1467–1480.e13.

Brangwynne, C.P., Eckmann, C.R., Courson, D.S., Rybarska, A., Hoege, C., Gharakhani, J., Jülicher, F., and Hyman, A.A. (2009). Germline P granules are liquid droplets that localize by controlled dissolution/condensation. Science *324*, 1729.

Bresnahan, W.A., Hultman, G.E., and Shenk, T. (2000). Replication of wild-type and mutant human cytomegalovirus in life-extended human diploid fibroblasts. J. Virol. *74*, 10816–10818.

Cho, W.-K., Spille, J.-H., Hecht, M., Lee, C., Li, C., Grube, V., and Cisse, I.I. (2018). Mediator and RNA polymerase II clusters associate in transcription-dependent condensates. Science *361*, 412.

Ciocco-Schmitt, G.M., Karabekian, Z., Godfrey, E.W., Stenberg, R.M., Campbell, A.E., and Kerry, J.A. (2002). Identification and Characterization of Novel Murine Cytomegalovirus M112–113 (e1) Gene Products. Virology *294*, 199–208.

Cohen, E.M., and Kobiler, O. (2016). Gene expression correlates with the number of herpes viral genomes initiating infection in single cells. Plos Pathog. *12*, e1006082.

Cubuk, J., Alston, J.J., Incicco, J.J., Singh, S., Stuchell-Brereton, M.D., Ward, M.D., Zimmerman, M.I., Vithani, N., Griffith, D., Wagoner, J.A., et al. (2021). The SARS-CoV-2 nucleocapsid protein is dynamic, disordered, and phase separates with RNA. Nat. Commun. *12*, 1936.

Dosztányi, Z., Csizmok, V., Tompa, P., and Simon, I. (2005). IUPred: web server for the prediction of intrinsically unstructured regions of proteins based on estimated energy content. Bioinformatics *21*, 3433–3434.

Du, M., and Chen, Z.J. (2018). DNA-induced liquid phase condensation of cGAS activates innate immune signaling. Science *361*, 704.

Dunn, W., Chou, C., Li, H., Hai, R., Patterson, D., Stolc, V., Zhu, H., and Liu, F. (2003). Functional profiling of a human cytomegalovirus genome. Proc. Natl. Acad Sci *100*, 14223–14228.

Everett, R.D. (1988). Analysis of the functional domains of herpes simplex virus type 1 immediate-early polypeptide Vmw110. J. Mol. Biol. *202*, 87.

Fei, J., Jadaliha, M., Harmon, T.S., Li, I.T.S., Hua, B., Hao, Q., Holehouse, A.S., Reyer, M., Sun, Q., Freier, S.M., et al. (2017). Quantitative analysis of multilayer organization of proteins and RNA in nuclear speckles at super resolution. J. Cell Sci. *130*, 4180–4192.

Feric, M., Vaidya, N., Harmon, T.S., Mitrea, D.M., Zhu, L., Richardson, T.M., Kriwacki, R.W., Pappu, R.V., and Brangwynne, C.P. (2016). Coexisting liquid phases underlie nucleolar subcompartments. Cell *165*, 1686–1697.

Fox, A.H., Nakagawa, S., Hirose, T., and Bond, C.S. (2018). Paraspeckles: where long noncoding RNA meets phase separation. Trends Biochem. Sci. *43*, 124–135.

Geiger, F., Acker, J., Papa, G., Wang, X., Arter, W.E., Saar, K.L., Erkamp, N.A., Qi, R., Bravo, J.P., Strauss, S., et al. (2021). Liquid–liquid phase separation underpins the formation of replication factories in rotaviruses. Embo J. *40*, e107711.

Griffiths, P., Baraniak, I., and Reeves, M. (2015). The pathogenesis of human cytomegalovirus. J. Pathol. *235*, 288–297.

Guseva, S., Milles, S., Jensen, M.R., Salvi, N., Kleman, J.-P., Maurin, D., Ruigrok, R.W.H., and Blackledge, M. (2020). Measles virus nucleo- and phosphoproteins form liquid-like phase-separated compartments that promote nucleocapsid assembly. Sci. Adv. *6*, eaaz7095.

Heinrich, B.S., Maliga, Z., Stein, D.A., Hyman, A.A., and Whelan, S.P.J. (2018). Phase transitions drive the formation of vesicular stomatitis virus replication compartments. MBio *9*, e02290–17.

Hyman, A.A., Weber, C.A., and Jülicher, F. (2014). Liquid-liquid phase separation in biology. Annu. Rev. Cell Developmental Biol. *30*, 39–58.

Iwayama, S., Yamamoto, T., Furuya, T., Kobayashi, R., Ikuta, K., and Hirai, K. (1994). Intracellular localization and DNA-binding activity of a class of viral early phosphoproteins in human fibroblasts infected with human cytomegalovirus (Towne strain). J. Gen. Virol. *75*, 3309–3318.

Kagele, D., Rossetto, C.C., Tarrant, M.T., and Pari, G.S. (2012). Analysis of the interactions of viral and cellular factors with human cytomegalovirus lytic origin of replication, oriLyt. Virology *424*, 106–114.

Kim, Y.E., and Ahn, J.H. (2010). Role of the specific interaction of UL112-113 p84 with UL44 DNA polymerase processivity factor in promoting DNA replication of human cytomegalovirus. J. Virol. *84*, 8409–8421.

Kim, Y.-E., Park, M.Y., Kang, K.J., Han, T.H., Lee, C.H., and Ahn, J.-H. (2015). Requirement of the N-terminal residues of human cytomegalovirus UL112-113 proteins for viral growth and oriLyt-dependent DNA replication. J. Microbiol. *53*, 561–569.

Kobiler, O., Lipman, Y., Therkelsen, K., Daubechies, I., and Enquist, L.W. (2010). Herpesviruses carrying a Brainbow cassette reveal replication and expression of limited numbers of incoming genomes. Nat. Commun. *1*, 146.

Kroschwald, S., Maharana, S., Mateju, D., Malinovska, L., Nüske, E., Poser, I., Richter, D., and Alberti, S. (2015). Promiscuous interactions and protein disaggregases determine the material state of stress-inducible RNP granules. ELife *4*, e06807.

Kroschwald, S., Maharana, S., and Simon, A. (2017). Hexanediol: A Chemical Probe to Investigate the Material Properties of Membrane-Less Compartments. Matters, e201702000010. https://doi.org/10.19185/matters.201702000010. http://sosjournals.s3.amazonaws.com/Qm4Tb429k12BV4sA.pdf.

Lee, D.S.W., Wingreen, N.S., and Brangwynne, C.P. (2021). Chromatin mechanics dictates subdiffusion and coarsening dynamics of embedded condensates. Nat. Phys. *17*, 531.

Li, M., Ball, C.B., Collins, G., Hu, Q., Luse, D.S., Price, D.H., and Meier, J.L. (2020). Human cytomegalovirus IE2 drives transcription initiation from a select subset of late infection viral promoters by host RNA polymerase II. PLoS Pathog. *16*, 1–30.

Lin, Y., Protter, D.S.W., Rosen, M.K., and Parker, R. (2015). formation and maturation of phase-separated liquid droplets by RNA-binding proteins. Mol. Cell *60*, 208–219.

McSwiggen, D.T., Mir, M., Darzacq, X., and Tjian, R. (2019a). Evaluating phase separation in live cells: diagnosis, caveats, and functional consequences. Genes Dev. *33*, 1619–1634.

McSwiggen, D.T., Hansen, A.S., Teves, S.S., Marie-Nelly, H., Hao, Y., Heckert, A.B., Umemoto, K.K., Dugast-Darzacq, C., Tjian, R., and Darzacq, X. (2019b). Evidence for DNA-mediated nuclear compartmentalization distinct from phase separation. ELife *8*, e47098.

CellPress
OPEN ACCESS

McVoy, M.A., and Adler, S.P. (1994). Human cytomegalovirus DNA replicates after early circularization by concatemer formation, and inversion occurs within the concatemer. J. Virol. *68*, 1040–1051.

Mochida, K., and Gomyoda, M. (1987). Toxicity of ethylene glycol, diethylene glycol, and propylene glycol to human cells in culture. B Environ. Contam. Tox. *38*, 151–153.

Muscolino, E., Schmitz, R., Loroch, S., Caragliano, E., Schneider, C., Rizzato, M., Kim, Y.-H., Krause, E., Lisnić, V.J., Sickmann, A., et al. (2020). Herpesviruses induce aggregation and selective autophagy of host signalling proteins NEMO and RIPK1 as an immune-evasion mechanism. Nat. Microbiol. *5*, 331–342.

Muscolino, E., Luoto, L.-M., and Brune, W. (2021). Viral induced protein aggregation: a mechanism of immune evasion. Int. J. Mol. Sci. *22*, 9624.

Nikolic, J., Bars, R.L., Lama, Z., Scrima, N., Lagaudrière-Gesbert, C., Gaudin, Y., and Blondel, D. (2017). Negri bodies are viral factories with properties of liquid organelles. Nat. Commun. *8*, 58.

Pari, G.S., and Anders, D.G. (1993). Eleven loci encoding trans-acting factors are required for transient complementation of human cytomegalovirus oriLyt-dependent DNA replication. J. Virol. *67*, 6979–6988.

Park, M.-Y., Kim, Y.-E., Seo, M.-R., Lee, J.-R., Lee, C.H., and Ahn, J.-H. (2006). Interactions among four proteins encoded by the human cytomegalovirus UL112-113 region regulate their intranuclear targeting and the recruitment of UL44 to prereplication foci. J. Virol. *80*, 2718–2727.

Patel, A., Lee, H.O., Jawerth, L., Maharana, S., Jahnel, M., Hein, M.Y., Stoynov, S., Mahamid, J., Saha, S., Franzmann, T.M., et al. (2015). A liquid-to-solid phase transition of the ALS protein FUS accelerated by disease mutation. Cell *162*, 1066–1077.

Pellet, P.E., and Roizman, B. (2007). Herpesviridae: a brief introduction, Sixth Edition. D.M. Knipe and P.M. Howley, eds., 1802–18022.

Penfold, M.E., and Mocarski, E.S. (1997). Formation of cytomegalovirus DNA replication compartments defined by localization of viral proteins and DNA synthesis. Virology *239*, 46–61.

Preston, C.M., Mabbs, R., and Nicholl, M.J. (1997). Construction and characterization of herpes simplex virus type 1 mutants with conditional defects in immediate early gene expression. Virology *229*, 228–239.

Riback, J.A., Zhu, L., Ferrolino, M.C., Tolbert, M., Mitrea, D.M., Sanders, D.W., Wei, M.-T., Kriwacki, R.W., and Brangwynne, C.P. (2020). Composition-dependent thermodynamics of intracellular phase separation. Nature *581*, 209–214.

Saar, K.L., Morgunov, A.S., Qi, R., Arter, W.E., Georg, K., Lee, A.A., and Knowles, T.P.J. (2021). Learning the molecular grammar of protein condensates from sequence determinants and embeddings. Proc. Natl. Acad. Sci. *118*, e2019053118.

Sampaio, K.L., Cavignac, Y., Stierhof, Y.-D., and Sinzger, C. (2005). Human cytomegalovirus labeled with green fluorescent protein for live analysis of intracellular particle movements. J. Virol. *79*, 2754–2767.

Sarisky, R., and Hayward, G. (1996). Evidence that the UL84 gene product of human cytomegalovirus is essential for promoting oriLyt-dependent DNA replication and formation of replication compartments in cotransfection assays. J. Virol. *70*, 7398–7413.

Scherer, M., and Stamminger, T. (2016). Emerging role of PML nuclear bodies in innate immune signaling. J. Virol. *90*, 5850–5854.

Schneider, C.A., Rasband, W.S., and Eliceiri, K.W. (2012). NIH Image to ImageJ: 25 years of image analysis. Nat. Methods *9*, 671–675.

Schommartz, T., Loroch, S., Alawi, M., Grundhoff, A., Sickmann, A., and Brune, W. (2016). Functional dissection of an alternatively spliced herpesvirus gene by splice site mutagenesis. J. Virol. *90*, 4626–4636.

Schommartz, T., Tang, J., Brost, R., and Brune, W. (2017). Differential requirement of human cytomegalovirus UL112-113 protein isoforms for viral replication. J. Virol. *91*, e00254-17.

Seyffert, M., Georgi, F., Tobler, K., Bourqui, L., Anfossi, M., Michaelsen, K., Vogt, B., Greber, U.F., and Fraefel, C. (2021). The HSV-1 transcription factor ICP4 confers liquid-like properties to viral replication compartments. Int. J. Mol. Sci. *22*, 4447.

Shevtsov, S.P., and Dundr, M. (2011). Nucleation of nuclear bodies by RNA. Nat. Cell Biol *13*, 167–173.

Shin, Y., Chang, Y.-C., Lee, D.S.W., Berry, J., Sanders, D.W., Ronceray, P., Wingreen, N.S., Haataja, M., and Brangwynne, C.P. (2018). Liquid nuclear condensates mechanically sense and restructure the genome. Cell *175*, 1481–1491.e13.

Silva, L.A., Loregian, A., Pari, G.S., Strang, B.L., and Coen, D.M. (2010). The carboxy-terminal segment of the human cytomegalovirus DNA polymerase accessory subunit UL44 is crucial for viral replication. J. Virol. *84*, 11563–11568.

Singatulina, A.S., Hamon, L., Sukhanova, M.V., Desforges, B., Joshi, V., Bouhss, A., Lavrik, O.I., and Pastré, D. (2019). PARP-1 activation directs FUS to DNA damage sites to form PARG-reversible compartments enriched in damaged DNA. Cell Rep. *27*, 1809–1821.e5.

Sinzger, C., Hahn, G., Digel, M., Katona, R., Sampaio, K.L., Messerle, M., Hengel, H., Koszinowski, U., Brune, W., and Adler, B. (2008). Cloning and sequencing of a highly productive, endotheliotropic virus strain derived from human cytomegalovirus TB40/E. J. Gen. Virol. *89*, 359–368.

Spector, D.J. (2015). UL84-independent replication of human cytomegalovirus strains conferred by a single codon change in UL122. Virology *476*, 345–354.

Strang, B.L. (2015). Viral and cellular subnuclear structures in human cytomegalovirus-infected cells. J. Gen. Virol. *96*, 239–252.

Strom, A.R., and Brangwynne, C.P. (2019). The liquid nucleome - phase transitions in the nucleus at a glance. J. Cell Sci *132*, jcs235093.

Tang, J., Brixel, R., and Brune, W. (2019). Copy-paste mutagenesis: a method for large-scale Alteration of viral genomes. Int. J. Mol. Sci. *20*, 913.

Taniguchi, T., Shimamoto, T., Isegawa, Y., Kondo, K., and Yamanishi, K. (2000). Structure of transcripts and proteins encoded by U79-80 of human herpesvirus 6 and its subcellular localization in infected cells. Virology *271*, 307–320.

Tischer, B.K., Smith, G.A., and Osterrieder, N. (2010). Vitro mutagenesis protocols, Third Edition. Methods Mol. Biol. *634*, 421–430.

Uebel, C., and Phillips, C. (2019). Phase-separated protein dynamics are affected by fluorescent tag choice. Micropublication Biol. *2019*, 143.

Vladimirova, O., Leo, A.D., Deng, Z., Wiedmer, A., Hayden, J., and Lieberman, P.M. (2021). Phase separation and DAXX redistribution contribute to LANA nuclear body and KSHV genome dynamics during latency and reactivation. Plos Pathog. *17*, e1009231.

Wells, R., Stensland, L., and Vieira, J. (2009). The human cytomegalovirus UL112-113 locus can activate the full Kaposi's sarcoma-associated herpesvirus lytic replication cycle. J. Virol. *83*, 4695–4699.

Wright, D., Staprans, S., and Spector, D. (1988). Four phosphoproteins with common amino termini are encoded by human cytomegalovirus AD169. J. Virol. *62*, 331–340.

Yu, D., Silva, M.C., and Shenk, T. (2003). Functional map of human cytomegalovirus AD169 defined by global mutational analysis. Proc. Natl. Acad. Sci. U S A *100*, 12396–12401.

Zhou, H., Song, Z., Zhong, S., Zuo, L., Qi, Z., Qu, L.-J., and Lai, L. (2019a). Mechanism of DNA-induced phase separation for transcriptional repressor VRN1. Angew. Chem. Int. Ed. *58*, 4858–4862.

Zhou, Y., Su, J.M., Samuel, C.E., Ma, D., and Dutch, R.E. (2019b). Measles virus forms inclusion bodies with properties of liquid organelles. J. Virol. *93*, e00948–19.

## STAR★METHODS

### KEY RESOURCES TABLE

| REAGENT or RESOURCE | SOURCE | IDENTIFIER |
|---|---|---|
| Antibodies | | |
| Mouse anti-mNeonGreen | Chromotek | RRID:AB_2827566 |
| Rat anti-BrdU | Abcam | RRID:AB_305426 |
| Mouse anti-UL44 (ICP36) | Virusys | Cat# CA006 |
| Goat anti-Mouse IgG Alexa 555 | Invitrogen | RRID:AB_2633276 |
| Goat anti-Mouse IgG Alexa 647 | Invitrogen | RRID:AB_2633277 |
| Goat anti-Rabbit IgG Alexa 647 | Invitrogen | RRID:AB_2633282 |
| Bacterial and virus strains | | |
| HCMV-NeonGreen-UL112 | This study | N/A |
| HCMV-UL32-GFP | Sampaio et al., 2005 | N/A |
| HSV-1 in1374 | Preston et al., 1997 | N/A |
| Chemicals, peptides, and recombinant proteins | | |
| Fibronectin bovine plasma | Sigma Aldrich | Cat# F1141 |
| Hoechst 33342 | Thermo Fisher | Cat# H1399 |
| Polyethylenimine (PEI), branched | Sigma Aldrich | Cat# 408727 |
| Doxycycline (Dox) Hyclate | LKT Labs | Cat# LKT- D5897.5 |
| Phosphonoacetic acid (PAA) | Sigma Aldrich | Cat# 284270 |
| Dulbecco's Modified Eagle's Medium (DMEM) with glucose | Sigma Aldrich | Cat# D5796 |
| Penicillin/streptomycin (100x) | Sigma Aldrich | Cat# P4333 |
| Fetal calf serum (FCS), tetracycline-free | Pan Biotech | Cat# P30-3602 |
| Fetal calf serum (FCS) | Pan Biotech | Cat# P30-0602 |
| 1,6-Hexanediol | Sigma Aldrich | Cat# 240117 |
| T4 ligase | New England Biolabs | Cat# M0202L |
| HisPur Ni-NTA Resin | Thermo Fisher | Cat# 88221 |
| Propylene glycol | Sigma Aldrich | Cat# 398039-25ML |
| 5-Bromo-2′-Deoxyuridine (BrdU) | Sigma Aldrich | Cat# B5002 |
| Experimental models: Cell lines | | |
| MRC-5 human embryonic lung fibroblasts | ATCC | RRID:CVCL_0440 |
| Telomerase-immortalized HFF | Bresnahan et al., 2000 | N/A |
| HEK-293T | ATCC | RRID:CVCL_1926 |
| HEK-293A | Invitrogen | RRID:CVCL_0045 |
| T-REx-293 | Invitrogen | RRID:CVCL_D585 |
| T-REx-293-mNeonGreen-UL112-113 | This study | N/A |
| Recombinant DNA | | |
| pcDNA3.1 | Thermo Fisher | Cat# V79020 |
| pcDNA mNeongreen-UL112-113 | This study | N/A |
| pcDNA mNeongreen | This study | N/A |
| pcDNA His-mNeongreen | This study | N/A |
| pcDNA M45-mCherry | Muscolino et al., 2020 | N/A |
| pmCherry-Nucleolin | Muscolino et al., 2020 | N/A |
| pEPkan-S2 | Addgene | RRID:Addgene_61601 |
| TB40-BAC4 | Sinzger et al., 2008 | N/A |
| TB40-BAC4-mNeonGreen-UL112 | This study | N/A |

(*Continued on next page*)

*Continued*

| REAGENT or RESOURCE | SOURCE | IDENTIFIER |
|---|---|---|
| pHR-SFFVp-NLS-iLID::EGFP::FTH1 | Addgene | RRID:Addgene_122147 |
| pHR-SFFVp-HNRPA1C::mCherry::SspB | Addgene | RRID:Addgene_122668 |
| pHR-SFFVp-mCherry::SspB | This study | N/A |
| pHR-SFFVp-p43ΔN::mCherry::SspB | This study | N/A |
| pHR-SFFVp-p84ΔN::mCherry::SspB | This study | N/A |
| pcDNA6/TR | Invitrogen | Cat# V1025-20 |
| pcDNA4/T0 | Invitrogen | Cat# V1020-20 |
| pcDNA4/T0 mNeongreen-UL112-113 | This study | N/A |
| pMD2.G | Addgene | RRID:Addgene_12259 |
| pMDLg/pRRE | Addgene | RRID:Addgene_12251 |
| pRSV-Rev | Addgene | RRID:Addgene_12253 |
| pSG5-p43 | Park et al., 2006 | N/A |
| pSG5-p84 | Park et al., 2006 | N/A |
| pmCherry-C1 | Clontech | Cat# V011976 |
| pcDNA3.1-UL44 | Schommartz et al., 2017 | N/A |
| pmCherry-UL112-113 | This study | N/A |
| pcDNA 3.1 UL112-113-mNeonGreen | This study | N/A |
| pmCherry-UL44 | This study | N/A |
| pmCherry-UL44ΔIDR | This study | N/A |
| pmCherry-UL44ΔN | This study | N/A |
| pmCherry-UL44ΔIDR+p43IDR | This study | N/A |
| Software and algorithms | | |
| ImageJ/Fiji | Laboratory for Optical and Computational Instrumentation, University of Wisconsin at Madison, Madison, Wisconsin, USA | RRID:SCR_003070 |
| NIS-Elements | Nikon | RRID:SCR_014329 |
| Graphpad Prism 5 | GraphPad Software Inc | RRID:SCR_002798 |
| IUpred | Dosztányi et al., 2005 | RRID:SCR_014632 |

## RESOURCE AVAILABILITY

### Lead contact

Further information and requests for resources and reagents should be directed to and will be fulfilled by the lead contact, Wolfram Brune (wolfram.brune@leibniz-hpi.de).

### Materials availability

All uniques/stable reagents generated in this study are available from the Lead contact.

### Data and code availability

- The datasets supporting the current study have not been deposited in a public repository but are available from the lead contact on request.
- The paper does not report original code.
- Any additional information required to reanalyze the data reported in this paper is available from the lead contact upon request.

## EXPERIMENTAL MODEL AND SUBJECT DETAILS

### Cells and viruses

T-REx-293-mNeonGreen-UL112 cells were generated by transfecting T-REx-293 with pcDNA4/T0 mNeonGreen-UL112 followed by selection with 200 μg/mL Zeocin for five days. All cells were grown in Dulbecco’s modified Eagle medium (DMEM) supplemented with 10% FCS, 100 U/mL penicillin, and 100 μg/mL streptomycin. All cells used in this study were tested regularly for mycoplasma contamination by PCR.

The BAC clone of HCMV strain TB40/E, TB40-BAC4 (Sinzger et al., 2008), was modified by *en passant* mutagenesis (Tischer et al., 2010). Fluorescent tagging of the UL112-113 proteins was done by inserting coding sequence of mNeonGreen and a flexible 6x GGS linker at the 5' end of the UL112 ORF. To do this, the I-SceI/kan cassette was PCR-amplified from pEPkan-S2 and inserted into the KpnI site of pcDNA-mNeonGreen-UL112. Flanking homologies for excision of the cassette by homologous recombination were introduced through the PCR primers. The mNeonGreen-kan cassette was PCR amplified with overhangs for homologous recombination and used for *en passant* mutagenesis of TB40-BAC4 in *E.coli* strain GS1783. Correct insertion of the mNeonGreen marker was verified by sequencing. To reconstitute infectious virus, MRC-5 cells were transfected with BAC DNA as described (Tang et al., 2019). Virus stocks were produced on MRC-5 cells, and titers were determined using the median tissue culture infective dose (TCID50) method.

### Plasmids, transfection, and transduction

pcDNA-mNeonGreen-UL112, mCherry-UL112-113 and UL112-113-mNeonGreen plasmids were constructed by Gibson assembly. UL112-113, mCherry or mNeonGreen sequences were PCR amplified and assembled in a linearized pcDNA3.1 vector. A disordered linker (6xGGS) was inserted between UL112-113 and the fluorescent tags to maintain flexibility. The mNeonGreen-UL112 was inserted between HindIII and XbaI sites of pcDNA4/TO (ThermoFisher). pHR-SFFVp-p43IDR::mCherry::sspB and pHR-SFFVp-p84IDR::mCherry::sspB were cloned by Gibson assembly. The UL112-113 p43 and p84 cDNA sequences lacking the first 75 nucleotides were PCR amplified from pDEST-SG5-HA-p43 and pDEST-SG5-HA-p84, respectively (kindly provided by Jin-Hyun Ahn, Sungkyunkwan University, Suwon, South Korea). The pHR-SFFVp-mCherry::sspB plasmid was generated by replacing HNRPA1C::mCherry with only mCherry. pmCherry-UL44, pmCherry-UL44ΔIDR, pmCherry-UL44ΔN, and pmCherry-UL44-ΔIDR+p43IDR were generated by Gibson assembly. The UL44 sequence was PCR amplified from pcDNA3.1-UL44 plasmid and inserted in pmCherry-C1. All newly generated plasmids were sequence-verified. HEK-293A, HEK-293T, and T-REx-293 cells were transfected by using polyethylenimine (PEI, Sigma). Lentivirus was produced in HEK-293T cells using standard protocols. Transduced cells were sorted on a FACS Aria III (BD Biosciences).

## METHODS DETAILS

### *In vitro* phase separation assay

mNeonGreen-labelled UL112-113 proteins were expressed in T-REx-293 mNeonGreen-UL112 cells induced with 2 μg/ml Dox. Three days after induction, cells were harvested, pelleted, and resuspended in a buffer containing 10 mM imidazole, 50 mM Tris–HCl (pH 7.4), 500 mM KCl and a cocktail of protease inhibitors. Cells were then lysed by three freeze-thaw cycles and sonicated 4 times (15 s each) at 5-10 W. Cell debris was removed by centrifugation (16.000 g for 30 min). The supernatant was incubated overnight with His-Pur Ni-NTA resin at 4°C. After several washing steps, proteins were eluted by adding 250 mM imidazole elution buffer (Thermo Fisher). Imidazole was removed using desalting columns (Sartorius). The proteins were resuspended in a 50 mM Tris–HCl (pH 7.4) buffer containing 500 mM KCl to prevent unwanted phase separation. For *in vitro* phase separation experiments, freshly prepared protein solution was centrifuged at high speed for two minutes to remove aggregates. Different protein concentrations were then mixed with buffers with increasing KCl concentration and plated on an 18-well slide (ibidi) and analyzed by microscopy.

### Hexanediol and propylene glycol treatment

1,6-Hexanediol powder was dissolved in complete DMEM and stored at 4°C. Propylene glycol was added directly to medium and stored at 4°C. For pulse experiments, cells were grown on a 35 mm dish (ibidi) coated with Fibronectin bovine plasma (Millipore). Cells were then treated with the desired compound concentration and the effect was assayed by direct visualization in live-cell imaging.

### EdU labeling

For production of EdU-labeled HCMV, MRC-5 cells were infected with mNeongreen-UL112-113 TB40/E (MOI = 1) and maintained in medium supplemented with 2 μM EdU (ThermoFisher). EdU-containing medium was replenished every day, and virus supernatant was harvested starting 4 dpi. Cell debris was removed by low-speed centrifugation. To remove unlabeled EdU, virions were pelleted by centrifugation (26.000 g for 2 h), resuspended in DMEM, loaded onto a 20% sucrose cushion, and pelleted again. Labeled virions were resuspended in DMEM with 10% FCS. For EdU pulse experiments, cells were infected at MOI 1 with an EdU-labeled virus. At the indicated time points, cells were incubated with medium containing 5 μM EdU prior to fixation.

### Western blotting

Western blots were performed according to standard protocols. In brief, proteins were denatured in SDS buffer, separated on SDS-PAGE, and transferred on a nitrocellulose membrane by semi-blot (Biorad). Membranes were successively stained by a primary (anti-mNeongreen, 32F6, Chromotek, 1:1000) and secondary antibody conjugated to horseradish peroxidase.For blue coomassie staining, gels were fixed in Coomassie fixing buffer (50% methanol,10% acetic acid, 40% water) for 30 min, stained in solution containing the 0.1% (v/v) of blue coomassie for 30 min and destained overnight in Coomassie destaining buffer (40% methanol, 10% acetic acid, 50% water).

## MICROSCOPY AND IMAGE ANALYSIS

### Microscopy modalities

Confocal laser scanning microscopy was performed with a Nikon TI2 based A1R confocal laser scanning unit equipped with PMT and GaAsP detectors, standard 404, 489, 561, and 637 laser lines, and corresponding filter sets. Nikon 1.4 NA 60x Plan Apo or a Nikon NA 0.9 40x objectives were used for image acquisition and pixel sizes were optimized for maximal resolution in NIS-Elements according to Nyquist. Spinning disk microscopy was carried out on a Nikon TI2 based spinning disk system equipped with a Yokogawa W2, a Nikon 1.49 NA Plan Apo objective, and an Andor iXON888 EMCCD. The resulting pixel size was 130 nm, and image acquisition was done with NIS-Elements. Further, the setup was equipped with 405, 488, 561, and 640 laser lines and corresponding filter sets. Widefield fluorescence microscopy was done on a Nikon TI based TIRF setup using a Nikon 60x 1.40 NA Plan-Apo objective and an Andor iXON897 EMCCD, equipped with 405, 488, 561, and 640 laser lines and corresponding filter sets. Life cell experiments on the different systems were carried out with identical humidified incubation chambers, heated to 37°C and 5% CO2 controlled by a gas mixer. Cells were grown in ibidi 35mm glass-bottom dishes coated with bovine plasma fibronectin.

### Fluorescence labeling

For immunofluorescence staining, cells were seeded on 8-well μ-slides (ibidi) one day before infection. Cells were then infected at a MOI of 1, and medium was changed 3 h later to remove excess virus. Cells were fixed with 4% PFA at different time points and permeabilized for 20 min in PBS containing 0.2% Triton X-100. After washing with PBS, cells were blocked using 3% bovine serum albumin (Sigma Aldrich) in PBS for 30 min, and subsequently incubated overnight at 4°C with primary antibodies. After washing, samples were incubated with 1:1000 diluted AlexaFluor-coupled secondary antibodies for 45 min at room temperature. Nuclei were stained using a 1:2000 Hoechst 33342 solution (ThermoFisher) for 10 min. To visualize EdU-labeled viral genomes, the Click-iT™ Plus EdU Cell Proliferation Kit for Imaging, with Alexa Fluor 555 (C10638, ThermoFisher) kit was used. Antibodies and antisera were used at the indicated concentrations: Anti-UL44 (1:1000), anti-mNeonGreen (1:1000), anti-BrdU (1:500), goat anti-mouse IgG Alexa 555 (1:2000), goat anti-mouse IgG Alexa 647 (1:2000), goat anti-rabbit IgG Alexa 647 (1:2000).

### Fluorescence recovery after photo bleaching and photoactivation

FRAP and photoactivation were done on a Nikon A1R confocal laser scanning microscope. For FRAP, an ROI of 1 μm was photobleached for 0.0625 s and the fluorescence intensity was recorded at two frames per second using NIS- element software (Nikon), for up to two minutes in total. For photobleaching, the same laser wavelength was used as for imaging. At least ten cells per condition were analyzed. Three areas were measured per cell over time. The bleached sample area (SA), the whole nucleus as reference (REF) and an area outside area that represented the background (BG) of the image were marked with regions of interest for quantification. Next, BG was subtracted from all corresponding measurements of SA and REF over time, resulting in SAcorr and REFcorr to correct for fluctuations in the illumination. Finally, general photobleaching due to the imaging was corrected by dividing all timepoints of SAcorr by their corresponding REFcorr values. The resulting ratio was then divided by the mean of the last ten timepoints of SA before photobleaching SA to calculate the degree of recovery over time (SAcorr/REFcorr)/SAinit) and plotted in Prism. To calculate the mobile fraction, we determined the recovery by first calculating SAfinal as the mean of SAcorr of the first 10 frames after fluorescence recovery had saturated and subtracted the intensity SAbleach which was the first measured frame after bleaching SA. We then divided the recovery by the amount of fluorescence drop as the difference between SAinit and SAbleach (SAfinal-SAbleach/SAinit-SAbleach). For calculating the recovery half-time, recovery curves were fitted to a nonlinear regression curve in GraphPad Prism version 5.0.0 for Windows, and Km was extracted.

Cells expressing iLiD and sspB constructs were globally photoactivated by imaging cells simultaneously with 488 and 563 nm laser illumination for 10 min. After 2 min, cells were imaged with only 563 nm to show the reversibility of the nucleation process. *In vivo* phase diagrams were plotted from fluorescence intensities of 150 cells per condition. For obtaining the core-to IDR ratio in the diluted phase, fluorescence intensities of the nucleoplasm were analyzed prior to photoactivation. The dense phase ratio was obtained by measuring fluorescence intensity in droplets after photoactivation. Local photoactivation was performed in cells that did not show clustering after 10 min of global activation. Local phase separation was induced by alternating 563 laser illumination for imaging and fast stimulation (0.125 s each) with 488 nm laser illumination at 0.01% laser power in a diffraction-limited spot.

### Fluorescence intensity profiles

Intensity profiles were measured in ImageJ (Schneider et al., 2012) by drawing a line across the relevant area and exporting the intensity measurements for each channel. The background measurement was performed by analyzing the mean fluorescence intensity of each channel in an area outside of the cell in the same image and its value was subtracted to all of the intensity measurements. The intensities were then normalized to a scale of 0–100% in GraphPad Prism and plotted against the position on the drawn line.

### EdU surface area measurements for quantification of viral genome replication and colocalization

EdU surface areas were quantified using ImageJ software. Manual ROIs were drawn around nuclei as identified by Hoechst staining and EdU-labeled areas were segmented by thresholding. Resulting EdU-ROIs were then used to measure signal intensities in the UL44 or BrdU channels where applicable.

## QUANTIFICATION AND STATISTICAL ANALYSIS

Data were analyzed using GraphPad Prism 5. Statistical significance was determined using unpaired Student t-tests. Plots show mean ± SD. (* $p < 0.05$, ** $p < 0.01$, *** $p < 0.001$).

**Cell Reports, Volume *38***

## Supplemental information

# Human cytomegalovirus forms phase-separated compartments at viral genomes to facilitate viral replication

**Enrico Caragliano, Stefano Bonazza, Giada Frascaroli, Jiajia Tang, Timothy K. Soh, Kay Grünewald, Jens B. Bosse, and Wolfram Brune**

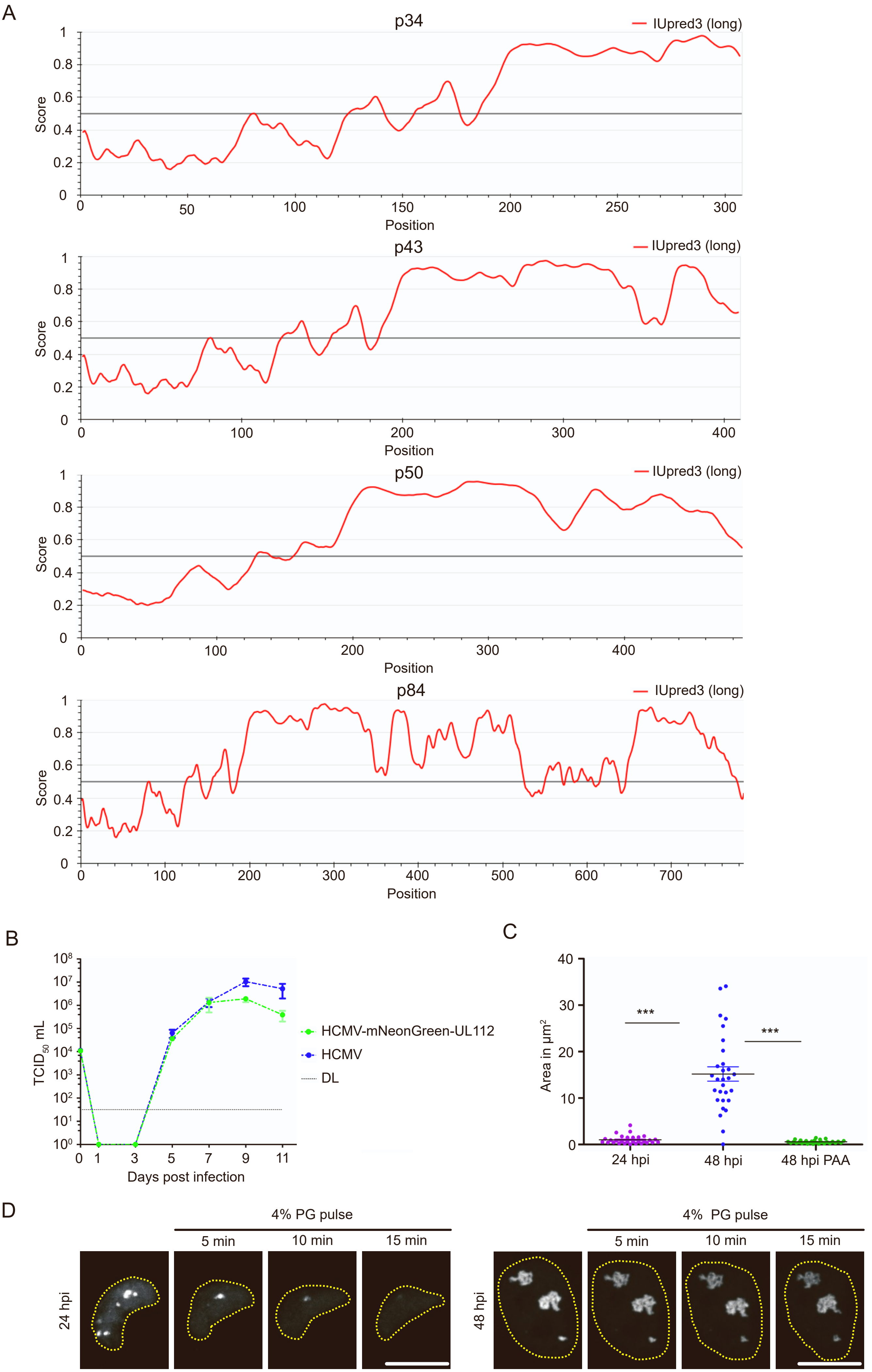
A
p34
IUpred3 (long)
Score
Position
p43
IUpred3 (long)
Score
Position
p50
IUpred3 (long)
Score
Position
p84
IUpred3 (long)
Score
Position
B
TCID50 mL
Days post infection
HCMV-mNeonGreen-UL112
HCMV
DL
C
Area in µm2
***
***
24 hpi
48 hpi
48 hpi PAA
D
4% PG pulse
5 min
10 min
15 min
24 hpi
4% PG pulse
5 min
10 min
15 min
48 hpi

**Supplemental Figure 1, related to Figure 1.**

(A) Disorder plots of the UL112-113 isoforms related to Fig 1. UL112-113 protein isoforms were analyzed by IUPred3 (Dosztányi et al., 2005) to predict disordered regions. The degree of disorder is scored on a scale from 0 to 1, with 1 indicating the highest level of disorder. Extended disordered regions are found in all four isoforms. The shared N-terminus is highly ordered.

(B) Replication kinetics of HCMV-mNeonGreen-UL112 and the parental HCMV strain. Cells were infected at an MOI of 0.1. Virus released into the supernatant was titrated.

(C) Quantification of the area of UL112-113 foci at 24 and 48 hpi or at 48 hpi in the presence of 250 µg/µl PAA.

(D) Treatment of infected cells with the LLPS inhibitor propylene glycol (PG, 4% w/v). MRC-5 cells were infected with HCMV-mNeonGreen-UL112 at an MOI of 1 for either 24 h or 48 h, treated with PG and imaged by live-cell spinning disk microscopy. Pictures show z-maximum intensity projection of 9 slices. The data shown are representative of three biological replicates (B) or three independent experiments (C-D). Yellow dotted lines indicate nuclear boundaries. Representative cells are shown in all panels.

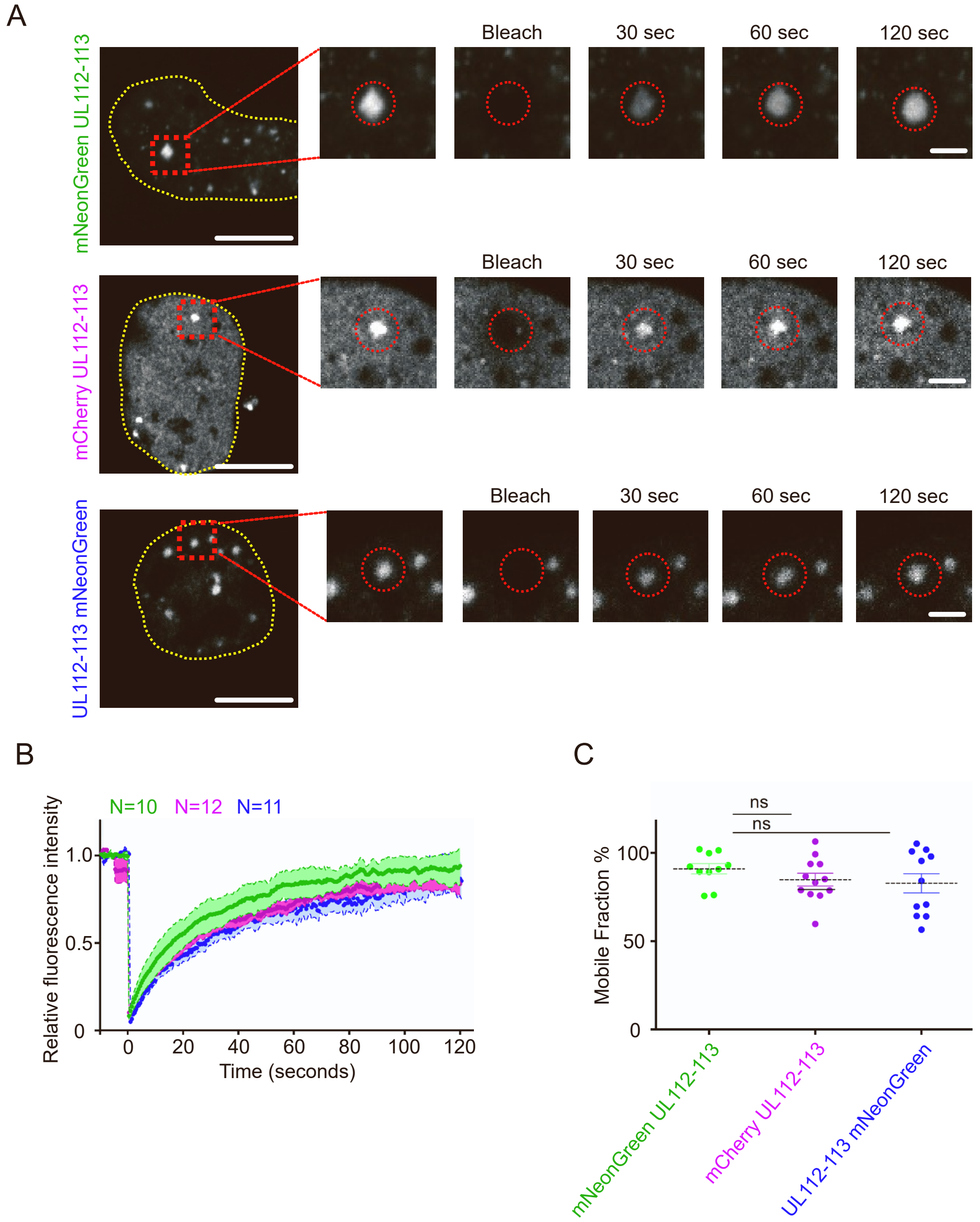


**Supplemental Figure 2, related to Figure 2.**

(A) Effect of tag and tag position on UL112-113 LLPS. HEK-293A cells were transfected with plasmids encoding mNeonGreen-UL112-113 (green), mCherry-UL112-113 (Magenta) or UL112-113-mNeonGreen (Blue). Cells were analyzed between 24 and 48 h post transfection. Scale bars indicate 10 µm and 2 µm in the insets.

(B) FRAP curves of transfected cells as described in (A). Curves represent an average of N different cells from 2 independent experiments.

(C) Mobile fractions from the cells analyzed in (B).

Yellow dotted lines indicate nuclear boundaries.

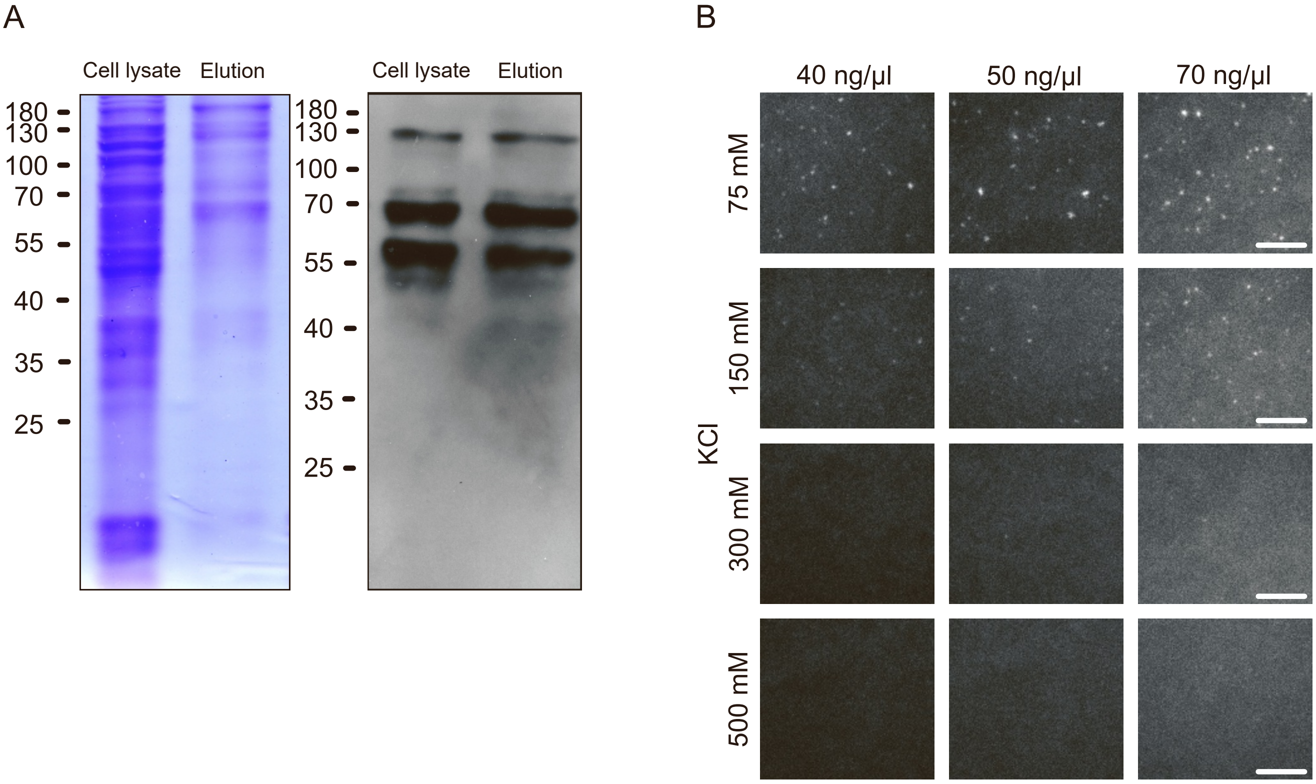


**Supplemental Figure 3, related to Figure 3.**

(A) Purification of UL112-113. mNeonGreen-UL112-113 expression was induced in T-REx-293 cells with 2 µg/ml Dox, and mNeonGreen-UL112-113 was purified with Ni-NTA beads. Proteins were separated on a 12% polyacrylamide gel. Protein purity was evaluated by Coomassie staining and western blot analysis. The four UL112-113 isoforms were detected with an mNeonGreen-specific antibody in the lysate (Cell lysate) and after elution from the beads (Elution).

(B) In vitro LLPS assay. A range of mNeonGreen-UL112-113 concentrations were mixed with a range of KCl buffers as indicated. Droplet formation was studied by widefield fluorescence microscopy.

(A-B) Data shown are representative of three independent experiments. Representative images are shown. Scale bar, 10 µm.

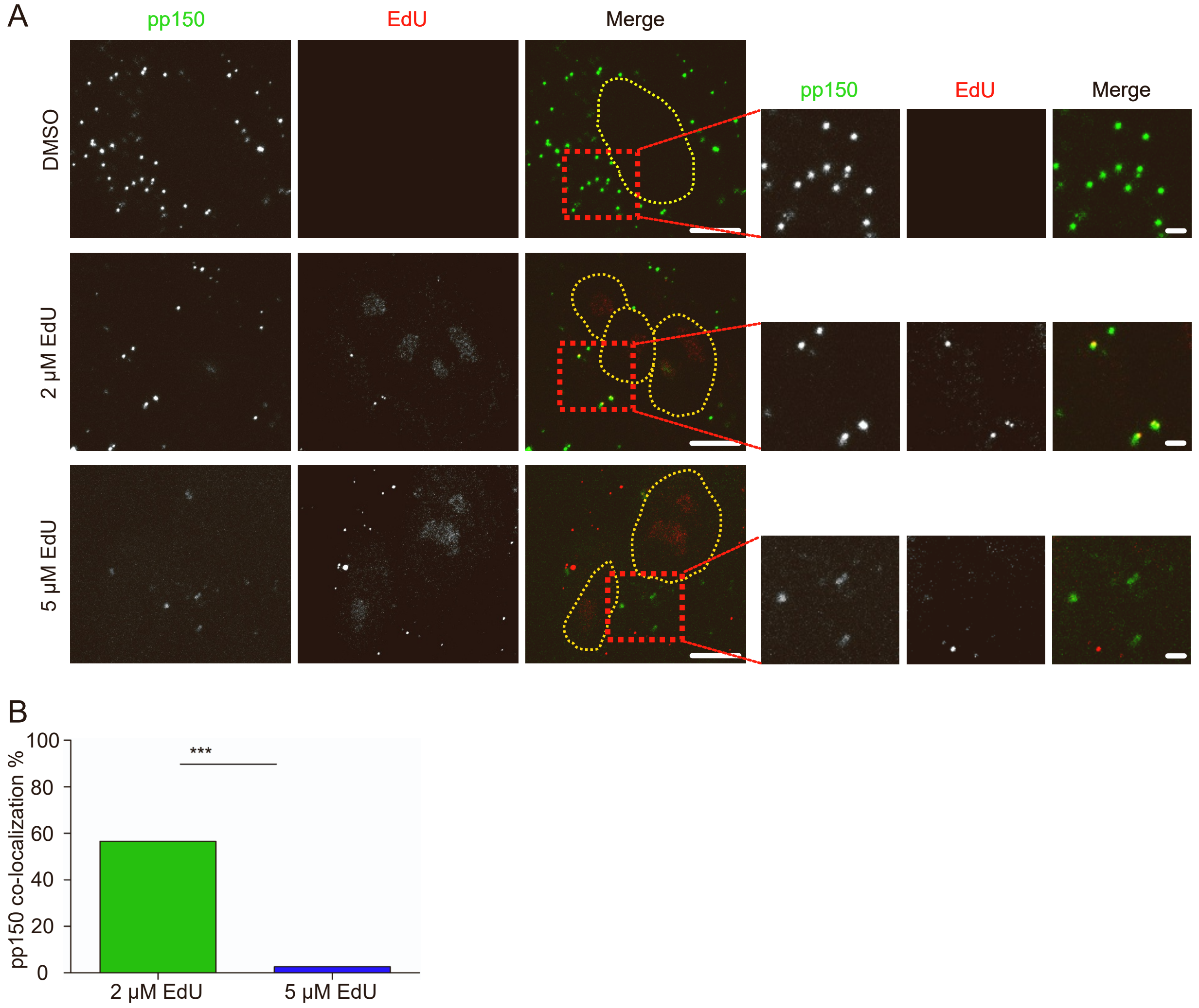


**Supplemental Figure 4, related to Figure 5**

(A) Association of capsids with viral genomes. HFF cells were infected (MOI 1) with HCMV-UL32-GFP that have been mock labeled (DMSO) or labeled with 2 or 5 µM EdU. UL32 encodes the capsid-associated inner tegument protein pp150. Two hpi cells were fixed and click-labeled with AF555-Picolyl-azide (EdU). Insets show EdU signals in relation to pp150-GFP labeled capsids.

(B) Quantification of genome association. Association of EdU labeled genomes with pp150-GFP capsids. The percentage capsids/ genome association is a representative example of two independent biological replicates. Yellow dotted lines indicate nuclear boundaries.

Representative images are shown. Scale bar, 10 µm.

A

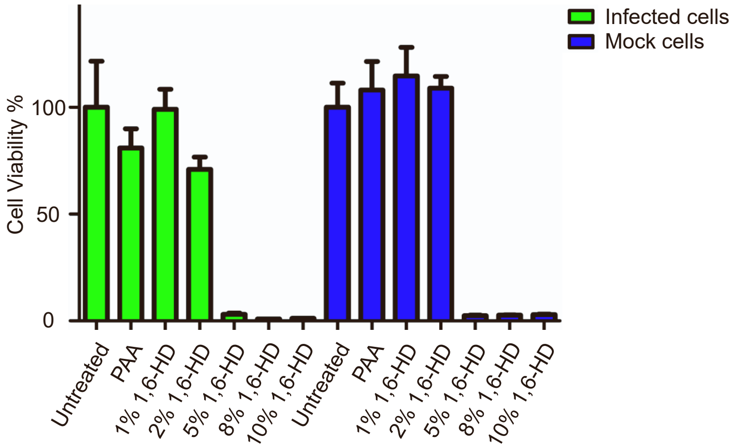


B

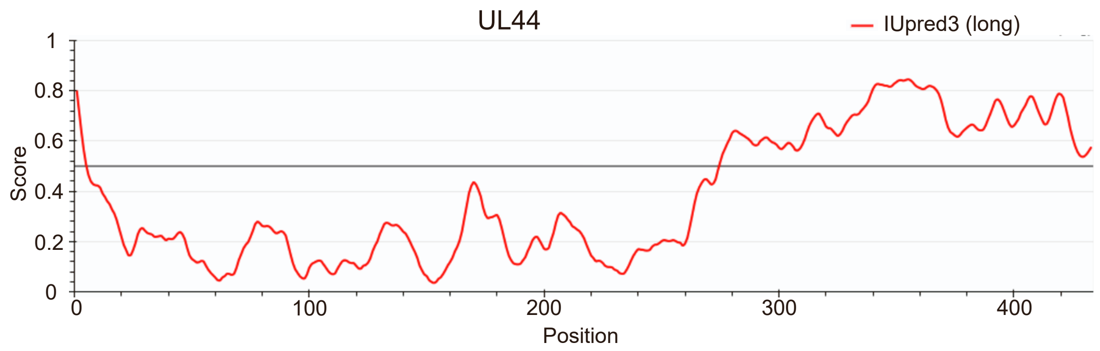


**Supplemental Figure 5, related to Figure 6**

(A) Cell viability after 1,6-HD treatment. Cells were infected at an MOI of 1 or mock infected. 24 hpi cells were treated with increasing amounts of 1,6-HD (1, 2, 5, 8, and 10%) or 250 µg/µl phosphonoacetic acid (PAA) for 2 hours. Cell viability was measured using an ATP assay. The percentage of cell viability is a representative example of three independent biological replicates.

(B) Disorder plot of the UL44 protein. The UL44 protein sequence was analyzed with IUPred3 (Dosztányi et al., 2005) to predict disordered regions. The degree of disorder is scored on a scale from 0 to 1, with 1 indicating the highest level of disorder. The N-terminus is predicted to be highly ordered, the C-terminus disordered.
